# Effects of ertugliflozin on kidney composite outcomes, renal function and albuminuria in patients with type 2 diabetes mellitus: an analysis from the randomised VERTIS CV trial

**DOI:** 10.1007/s00125-021-05407-5

**Published:** 2021-03-04

**Authors:** David Z. I. Cherney, Bernard Charbonnel, Francesco Cosentino, Samuel Dagogo-Jack, Darren K. McGuire, Richard Pratley, Weichung J. Shih, Robert Frederich, Mario Maldonado, Annpey Pong, Christopher P. Cannon, on behalf of the VERTIS CV Investigators

**Affiliations:** 1 University of Toronto, Toronto, ON Canada; 2 University of Nantes, Nantes, France; 3 Unit of Cardiology, Karolinska Institute & Karolinska University Hospital, Stockholm, Sweden; 4 University of Tennessee Health Science Center, Memphis, TN USA; 5 University of Texas Southwestern Medical Center, Dallas, TX USA; 6 Parkland Health and Hospital System, Dallas, TX USA; 7 AdventHealth Translational Research Institute, Orlando, FL USA; 8 Rutgers School of Public Health, New Brunswick, NJ USA; 9 Rutgers Cancer Institute of New Jersey, New Brunswick, NJ USA; 10 Pfizer Inc., Collegeville, PA USA; 11 MSD Limited, London, UK; 12 Merck & Co., Inc., Kenilworth, NJ USA; 13 Cardiovascular Division, Brigham and Women’s Hospital, Harvard Medical School, Boston, MA USA

**Keywords:** Cardiovascular disease, Diabetic nephropathies, Ertugliflozin, Type 2 diabetes mellitus

## Abstract

**Aims/hypothesis:**

In previous work, we reported the HR for the risk (95% CI) of the secondary kidney composite endpoint (time to first event of doubling of serum creatinine from baseline, renal dialysis/transplant or renal death) with ertugliflozin compared with placebo as 0.81 (0.63, 1.04). The effect of ertugliflozin on exploratory kidney-related outcomes was evaluated using data from the eValuation of ERTugliflozin effIcacy and Safety CardioVascular outcomes (VERTIS CV) trial (NCT01986881).

**Methods:**

Individuals with type 2 diabetes mellitus and established atherosclerotic CVD were randomised to receive ertugliflozin 5 mg or 15 mg (observations from both doses were pooled), or matching placebo, added on to existing treatment. The kidney composite outcome in VERTIS CV (reported previously) was time to first event of doubling of serum creatinine from baseline, renal dialysis/transplant or renal death. The pre-specified exploratory composite outcome replaced doubling of serum creatinine with sustained 40% decrease from baseline in eGFR. In addition, the impact of ertugliflozin on urinary albumin/creatinine ratio (UACR) and eGFR over time was assessed.

**Results:**

A total of 8246 individuals were randomised and followed for a mean of 3.5 years. The exploratory kidney composite outcome of sustained 40% reduction from baseline in eGFR, chronic kidney dialysis/transplant or renal death occurred at a lower event rate (events per 1000 person-years) in the ertugliflozin group than with the placebo group (6.0 vs 9.0); the HR (95% CI) was 0.66 (0.50, 0.88). At 60 months, in the ertugliflozin group, placebo-corrected changes from baseline (95% CIs) in UACR and eGFR were −16.2% (−23.9, −7.6) and 2.6 ml min^−1^ [1.73 m]^−2^ (1.5, 3.6), respectively. Ertugliflozin was associated with a consistent decrease in UACR and attenuation of eGFR decline across subgroups, with a suggested larger effect observed in the macroalbuminuria and Kidney Disease: Improving Global Outcomes in Chronic Kidney Disease (KDIGO CKD) high/very high-risk subgroups.

**Conclusions/interpretation:**

Among individuals with type 2 diabetes and atherosclerotic CVD, ertugliflozin reduced the risk for the pre-specified exploratory composite renal endpoint and was associated with preservation of eGFR and reduced UACR.

**Trial registration:**

ClinicalTrials.gov NCT01986881

**Graphical abstract:**

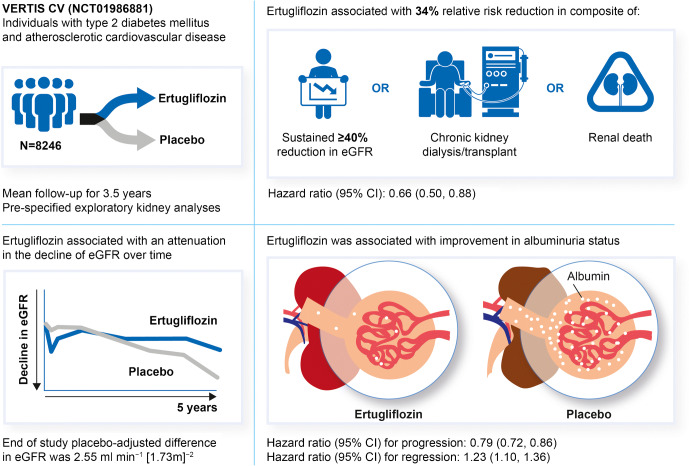

**Supplementary Information:**

The online version contains peer-reviewed but unedited supplementary material available at 10.1007/s00125-021-05407-5.

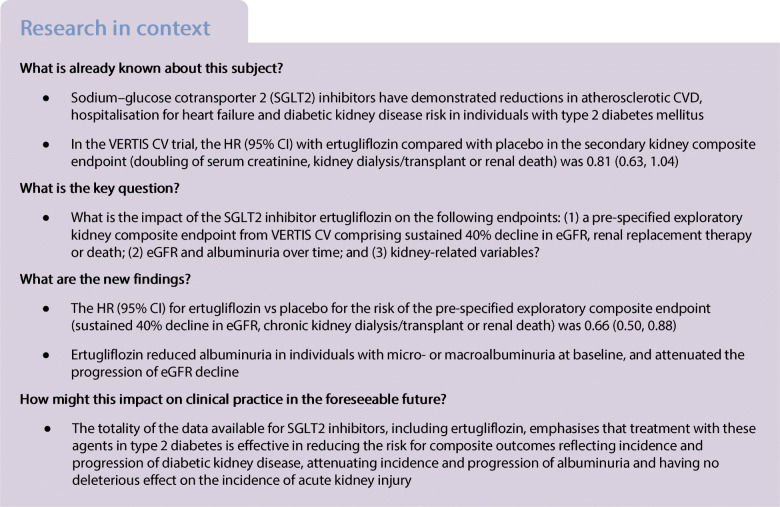



## Introduction

Ertugliflozin is a selective inhibitor of sodium–glucose cotransporter 2 (SGLT2), leading to glucosuria, with HbA_1c_ lowering of 8.3–9.9 mmol/mol (0.8–0.9%) and 2–3 kg of weight loss, and is approved for use as a glucose-lowering therapy in type 2 diabetes mellitus [1]. SGLT2 inhibitors induce other physiological effects, including natriuresis, contributing to lowering of BP and beneficial effects on kidney function [2]. Although glucosuria- and HbA_1c_-lowering effects attenuate as eGFR decreases below a certain threshold [3, 4], other effects (such as reductions in BP and weight) of SGLT2 inhibitors tend to be preserved across the range of eGFR that has been studied in humans [5].

In addition to lowering the urinary albumin/creatinine ratio (UACR) [6], SGLT2 inhibitors reduce the risk of the kidney composite, which includes a substantial decrease in renal filtering capacity (doubling of serum creatinine or a sustained 40% decrease in eGFR), renal replacement therapy and renal death, in cardiovascular outcome trials (CVOTs) in individuals with type 2 diabetes [7–9] and in individuals with established diabetic kidney disease (DKD) and macroalbuminuria [10]. In the eValuation of ERTugliflozin effIcacy and Safety CardioVascular outcomes (VERTIS CV) trial, the key secondary composite kidney endpoint (time to first occurrence of the composite of doubling of baseline serum creatinine, renal death, kidney dialysis/transplant) was not significantly reduced, with a HR (95% CI) of 0.81 (0.63, 1.04). The impact of ertugliflozin on kidney function with a definition used in other outcome studies [7, 9], such as sustained 40% decline in eGFR, has not yet been reported.

In the present analyses, our aim was to explore the effects of ertugliflozin on pre-specified exploratory kidney endpoints in the overall VERTIS CV trial population and according to baseline kidney function status, and to evaluate the effect of ertugliflozin on the incidence of acute kidney failure-related adverse events.

## Methods

### Study design and oversight

The design, primary results and full study protocol (protocol MK-8835-004) of the VERTIS CV trial have been previously published [11, 12]. The VERTIS CV trial (ClinicalTrials.gov registration no. NCT01986881) was a prospective, multicentre, randomised, double-blind, placebo-controlled, parallel-group, event-driven trial in individuals with type 2 diabetes and established atherosclerotic CVD comparing two doses of ertugliflozin (prospectively planned to be pooled for analyses) vs placebo. VERTIS CV was initiated in 2013. On the basis of evolving knowledge of the potential role of SGLT2 inhibitors in reducing the risk of cardiovascular events, the study was amended in 2015 to increase participant numbers so that these potential benefits could be adequately explored. The statistical analysis plan for the exploratory kidney outcomes was completed prior to database lock, before the results were known (see electronic supplementary material [ESM] Statistical analysis plan); thus, these are pre-specified analyses.

### Study population

The full details of trial eligibility criteria have been previously described [11, 12]. The trial recruited individuals with type 2 diabetes and established atherosclerotic CVD, with baseline eGFR ≥30 ml min^−1^ [1.73 m]^−2^. The trial was conducted in accordance with the principles of Good Clinical Practice and was approved by the appropriate institutional review boards and regulatory agencies, with all participants providing written informed consent.

### Classification by baseline kidney category

Three baseline kidney function classification schemes were pre-specified and used for subgroup analyses: (1) eGFR; (2) UACR; and (3) the Kidney Disease: Improving Global Outcomes in Chronic Kidney Disease (KDIGO CKD) risk categories, which combine eGFR and UACR. For the eGFR category, baseline eGFR was calculated using the Modification of Diet in Renal Disease (MDRD) study equation [13]; participants were classified into chronic kidney disease (CKD) stage 1, stage 2 or stage 3 if eGFR was ≥90 ml min^−1^ [1.73 m]^−2^, ≥60 ml min^−1^ [1.73 m]^−2^ and <90 or <60 ml min^−1^ [1.73 m]^−2^, respectively. For the classification by baseline UACR, participants were classified into normoalbuminuria, microalbuminuria or macroalbuminuria categories if UACR (mg/mmol [mg/g]) was <3.39 (<30), ≥3.39 and ≤33.9 (≥30 and ≤300) or >33.9 (>300), respectively. The KDIGO CKD risk category (low, moderate and high/very high risk) was based on the prognosis of CKD by eGFR (calculated with the MDRD equation) and albuminuria categories heat map (ESM Fig. 1) [14]. Individuals assigned to the KDIGO CKD high- and very high-risk categories were pooled for the analyses. For classification, a baseline eGFR and/or UACR value was required. For the analyses of eGFR over time in the overall population, both the MDRD and Chronic Kidney Disease Epidemiology Collaboration (CKD-EPI) equations were used; the CKD-EPI equation was used for the analyses of eGFR over time in subgroups, as this formula has superior operating characteristics in terms of precision and accuracy and is more frequently used in clinical practice.

### Key kidney outcomes

All analyses reported here were pre-specified, either in the statistical analysis plan of the trial or in a separate analysis plan completed prior to database lock and unblinding (see ESM Statistical analysis plan). Briefly, results are reported for the hierarchically tested secondary composite kidney outcome (doubling of baseline serum creatinine, kidney dialysis/transplant or renal death), as well as the individual components of the composite, and a sensitivity analysis based on sustained doubling of serum creatinine (defined as the occurrence of a value that met the cut-off criterion that was followed, more than 30 days later, by a subsequent value that also met the cut-off criterion), chronic kidney dialysis/transplant or renal death. As a sustained 40% decline from baseline in eGFR is an appropriate surrogate endpoint for kidney failure that allows for the accrual of more events in a shorter period of time [15], we carried out a pre-specified exploratory composite kidney outcome analysis of a sustained 40% reduction from baseline in eGFR (defined as the occurrence of a value that met the cut-off criterion that was followed, more than 30 days later, by a subsequent value that also met the cut-off criterion), chronic kidney dialysis/transplant or renal death. The pre-specified exploratory composite kidney endpoint analysis was performed in the pooled ertugliflozin population and by ertugliflozin dose (5 mg and 15 mg). Changes in albuminuria over time and shifts in albuminuria status, as well as eGFR over time by treatment group, are also reported. Multiple events occurring in the same individual were counted on the first event only. All analyses were carried out for the overall population and in subgroups defined by baseline kidney function. The incidence of adverse events related to acute kidney failure was evaluated using the Acute Renal Failure Standard Medical Dictionary for Regulatory Activities Query (SMQ) in the overall population and by two baseline eGFR subgroups (eGFR <60 ml min^−1^ [1.73 m]^−2^ and eGFR ≥60 ml min^−1^ [1.73 m]^−2^).

### Statistical analysis

The analyses of kidney composite endpoints and each individual component were performed on the intention-to-treat population (all randomised participants). The analyses of eGFR and UACR endpoints were carried out on the full analysis set (FAS; randomised participants who received one or more doses of blinded study medication and had one or more measurements of the analysis endpoint). The analyses of eGFR and UACR endpoints were performed on the pooled ertugliflozin population and by ertugliflozin dose (5 mg and 15 mg). Data after the initiation of glycaemic rescue therapy were included; however, data obtained more than 2 days after the last dose of study medication were excluded from the change-from-baseline analyses of the eGFR and UACR endpoints. The analyses of the adverse event of acute kidney failure were carried out on the safety population (randomised participants who took one or more doses of study medication); adverse events occurring up to 30 days after the final dose of study medication were included.

The time-to-event endpoints were analysed using a stratified Cox proportional hazards model, including treatment group as a covariate with cohort as a stratification factor (cohort one [participants randomised before protocol amendment, between December 2013 and July 2015] and cohort two [participants randomised after protocol amendment, in 2016 and beyond]). For baseline subgroup analyses, the models included terms for treatment, subgroup (categorical) and treatment-by-subgroup interaction. The baseline subgroups were evaluated by eGFR category, KDIGO CKD risk categories and UACR category. The *p* value of the interaction term is presented for subgroup analyses. Time-to-event endpoints, if no event had occurred, were censored at the time of the last follow-up visit.

Mean changes from baseline in eGFR and UACR endpoints over time were estimated using the constrained longitudinal data analysis (cLDA) model for the overall population. The model contained the fixed effects for treatment, time, treatment-by-time interaction, baseline HbA_1c_ and baseline systolic BP (SBP) if the analysis model converged. Owing to the non-normal distribution of UACR, UACR data were log-transformed prior to analysis. The geometric means and adjusted mean percentage change (derived from exponentiation of adjusted estimates from the cLDA model) with 95% CIs are presented by treatment and timepoint. The difference between ertugliflozin treatment and placebo in mean percentage change in UACR from baseline was estimated and presented. For subgroup analyses of eGFR and UACR, a repeated measures ANCOVA (RMANCOVA) method was used. The RMANCOVA model adjusted for baseline of response variable, baseline HbA_1c_, treatment, time, subgroup, treatment-by-subgroup interaction and treatment-by-subgroup-by-time interaction. For subgroup analyses based on factors that were already in the main model, the respective term appeared in the model only once. In both cLDA and RMANCOVA models, time was treated as a categorical variable. An unstructured covariance matrix was used to model the correlation among repeated measurements. When the covariance structure did not converge, a Toeplitz covariance structure was used. Summary statistics are provided for baseline demographic and disease characteristics. In this exploratory kidney outcomes analysis, type 1 error of 5% was not controlled for multiple testing. The nominal *p* values are reported. All statistical analyses were performed using SAS Version 9.4 (The SAS Institute, Cary, NC, USA).

## Results

### Baseline characteristics

A total of 8246 individuals were randomised and followed for a mean (SD) of 3.5 (1.2) years. Of these, 2747 received placebo and 5499 received ertugliflozin (5 mg or 15 mg doses). Details of participant disposition and study conduct have been reported [12]. Of the randomised individuals, 87–88% completed the trial alive, 8.5–9.2% died and 3.7–4.1% withdrew. Study medication was discontinued prematurely in 27.9% and 23.5% of participants in the placebo and ertugliflozin groups, respectively. The median duration of follow-up was 3.0 years. Table 1 summarises the baseline characteristics in the overall population. In the overall cohort, 2048 (24.8%), 4390 (53.2%) and 1807 (21.9%) individuals had CKD stage 1, 2 and 3 at baseline, respectively. At baseline, 4783 (59.6%), 2492 (31.0%) and 755 (9.4%) individuals exhibited normoalbuminuria, microalbuminuria and macroalbuminuria, respectively. The number of participants assigned to the KDIGO CKD low-risk, moderate-risk and high-/very high-risk categories at baseline was 3916 (48.8%), 2568 (32.0%) and 1548 (19.3%), respectively. At baseline, the mean age of participants was 64.4 years, mean duration of type 2 diabetes was 13.0 years, mean HbA_1c_ was 66.5 mmol/mol (8.2%) and mean SBP was 133.3 mmHg.

**Table 1:** Baseline demographic and disease characteristics of the overall population (ITT)

Characteristic	Placebo (*n* = 2747)	Ertugliflozin, pooled (*n* = 5499)	Total (*N* = 8246)
Female sex, *n* (%)	844 (30.7)	1633 (29.7)	2477 (30.0)
Age, years	64.4 ± 8.0	64.4 ± 8.1	64.4 ± 8.1
HbA_1c_, mmol/mol	66.3 ± 10.3	66.6 ± 10.5	66.5 ± 10.4
HbA_1c_, %	8.2 ± 0.9	8.2 ± 1.0	8.2 ± 1.0
Duration of T2DM, years	13.1 ± 8.4	12.9 ± 8.3	13.0 ± 8.3
Haemoglobin, g/l	139.5 ± 13.7	140.0 ± 13.5	139.9 ± 13.6
BMI, kg/m^2^	32.0 ± 5.5	31.9 ± 5.4	32.0 ± 5.4
eGFR, ml min^−1^ [1.73 m]^−2^ (MDRD)	75.7 ± 20.8	76.1 ± 20.9	76.0 ± 20.9
UACR, mg/mmol	2.1 (0.7–7.5)	2.0 (0.7–7.8)	2.1 (0.7–7.7)
UACR, mg/g	19.0 (6.0–66.5)	18.0 (6.0–69.0)	19.0 (6.0–68.0)
SBP, mmHg	133.1 ± 13.9	133.5 ± 13.7	133.3 ± 13.8
Glucose-lowering agents, *n* (%)			
Insulin	1344 (48.9)	2556 (46.5)	3900 (47.3)
Biguanides	2124 (77.3)	4168 (75.8)	6292 (76.3)
Antihypertensive agents, *n* (%)			
Any antihypertensive	2632 (95.8)	5221 (94.9)	7853 (95.2)
RAAS inhibitor	2239 (81.5)	4447 (80.9)	6686 (81.1)
Diuretic	1196 (43.5)	2346 (42.7)	3542 (43.0)
Loop diuretic	426 (15.5)	826 (15.0)	1252 (15.2)
Mineralocorticoids receptor antagonists	224 (8.2)	450 (8.2)	674 (8.2)
Antiplatelet or antithrombotic drugs, *n* (%)	2446 (89.0)	4880 (88.7)	7326 (88.8)
Lipid-lowering agents, *n* (%)	2313 (84.2)	4655 (84.7)	6968 (84.5)
eGFR category, *n* (%)^a^			
CKD stage 1	678 (24.7)	1370 (24.9)	2048 (24.8)
CKD stage 2	1461 (53.2)	2929 (53.3)	4390 (53.2)
CKD stage 3	608 (22.1)	1199 (21.8)	1807 (21.9)
UACR category, *n* (%)^b^			
Normoalbuminuria	1597 (59.5)	3186 (59.6)	4783 (59.6)
Microalbuminuria	845 (31.5)	1647 (30.8)	2492 (31.0)
Macroalbuminuria	242 (9.0)	513 (9.6)	755 (9.4)
KDIGO CKD risk category, *n* (%)^c^			
Low risk of CKD	1307 (48.7)	2609 (48.8)	3916 (48.8)
Moderate risk of CKD	859 (32.0)	1709 (31.9)	2568 (32.0)
High/very high risk of CKD	517 (19.3)	1031 (19.3)	1548 (19.3)

Values are mean±SD or median (IQR) unless otherwise stated

^a^Participants required a baseline eGFR value for classification: *n* = 2747 for placebo; *n* = 5498 for ertuglifozin, pooled; *n* = 8245 total

^b^Participants required a baseline UACR value for classification: *n* = 2684 for placebo; *n* = 5346 for ertugliflozin, pooled; *n* = 8030 total

^c^Participants required baseline eGFR and UACR values for classification: *n* = 2683 for placebo; *n* = 5349 for ertuglifozin, pooled; *n* = 8032 total

ITT, intention to treat; RAAS, renin–angiotensin–aldosterone system; T2DM, type 2 diabetes mellitus

At baseline, mean eGFR was 76.0 ml min^−1^ [1.73 m]^−2^ and the median for UACR was 2.1 mg/mmol (19.0 mg/g) in the overall cohort. Baseline characteristics in the pooled ertugliflozin population were similar to those of the individual ertugliflozin 5 mg and 15 mg groups (ESM Table 1). Baseline clinical variables, including mean eGFR and median UACR, by the three kidney function categories are shown in ESM Tables 2–4.

### Pre-specified exploratory composite kidney outcomes

**Fig. 1:**
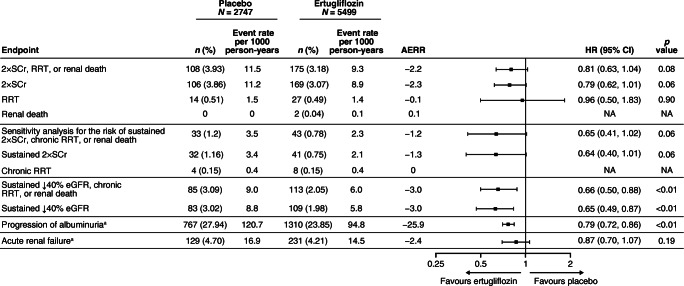
Forest plot of the key kidney outcomes in the overall population (ITT). ↓40% eGFR, 40% decline in eGFR; 2 × SCr, doubling of serum creatinine; AERR, absolute event risk reduction; ITT, intention to treat; RRT, renal replacement therapy (kidney dialysis or transplant). ^a^The analysis was performed on the full analysis set population: placebo, *n* = 2745; ertugliflozin, *n* = 5493

The hierarchically tested secondary composite kidney endpoint in VERTIS CV (time to first occurrence of the composite of doubling of baseline serum creatinine, kidney dialysis/transplant or renal death) occurred at an event rate (events per 1000 person-years) of 9.3 and 11.5 in the ertugliflozin and placebo groups, respectively, with a HR (95% CI) of 0.81 (0.63, 1.04) (Fig. 1), as previously reported [12]. The RR for the kidney composite endpoint with ertugliflozin compared with placebo was generally similar across subgroups defined by baseline kidney function (*p* > 0.05 for interaction; ESM Table 5). For the sensitivity analysis, the risk of the composite of sustained doubling of serum creatinine, chronic kidney dialysis/transplant or renal death occurred less frequently at an event rate (events per 1000 person-years) of 2.3 in the ertugliflozin group and 3.5 in the placebo group, with a HR (95% CI) of 0.65 (0.41, 1.02) (Fig. 1 and ESM Fig. 2). Similar results are reported in subgroups defined by baseline kidney function compared with the overall cohort (ESM Table 6).

**Fig. 2:**
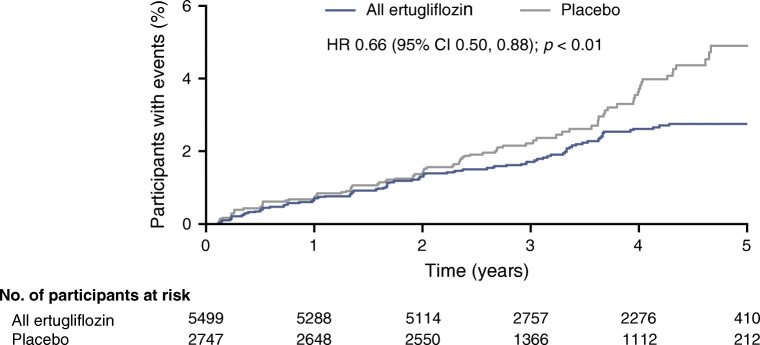
Kaplan–Meier plot for the time to first event in the pre-specified exploratory kidney composite outcome (sustained 40% decrease from baseline in eGFR, chronic renal dialysis/transplant or renal death) in the overall population (ITT). ITT, intention to treat

The pre-specified exploratory kidney composite outcome of sustained 40% reduction from baseline in eGFR, chronic kidney dialysis/transplant or renal death occurred at a lower event rate (events per 1000 person-years) in the ertugliflozin group than with placebo (6.0 vs 9.0), with a HR (95% CI) of 0.66 (0.50, 0.88) (Figs 1, 2). Similar results were obtained by ertugliflozin dose compared with the pooled ertugliflozin group, with HRs (95% CI) of 0.60 (0.42, 0.84) and 0.73 (0.53, 1.01) in the ertugliflozin 5 mg and ertugliflozin 15 mg groups, respectively (ESM Fig. 3). The lower rate of the pre-specified exploratory composite outcome in the ertugliflozin group compared with the placebo group was due to a lower rate of sustained 40% reduction from baseline in eGFR in the ertugliflozin group (HR [95% CI]: 0.65 [0.49, 0.87]) (Fig. 1).

The risk reduction for the pre-specified exploratory kidney composite endpoint with ertugliflozin compared with placebo is presented in ESM Table 7. There was large overlap of the 95% CIs, and the *p* values for interaction by baseline kidney function categories were non-significant.

### Progression or regression in albuminuria category

In the overall population, ertugliflozin was associated with a reduced risk of progression to microalbuminuria (in participants with normoalbuminuria at baseline) or macroalbuminuria (in those with normoalbuminuria or microalbuminuria at baseline) compared with placebo (Fig. 1). Event rates for albuminuria progression per 1000 person-years were 94.8 and 120.7 in the ertugliflozin and placebo groups, respectively, with a HR (95% CI) of 0.79 (0.72, 0.86).

In the overall population, ertugliflozin was associated with an increased regression to microalbuminuria (in participants with macroalbuminuria at baseline) or normoalbuminuria (in those with microalbuminuria or macroalbuminuria at baseline) compared with placebo. The event rate for regression of microalbuminuria and macroalbuminuria per 1000 person-years was 87.7 and 72.3 in the ertugliflozin and placebo group, respectively, with a HR (95% CI) of 1.23 (1.10, 1.36). While beneficial effects on regression of albuminuria were observed consistently among all subgroups, a larger effect is suggested in the subgroup with greater baseline elevation of albuminuria, as the *p* value for interaction was 0.04 (ESM Table 8).

### UACR change over time

**Fig. 3:**
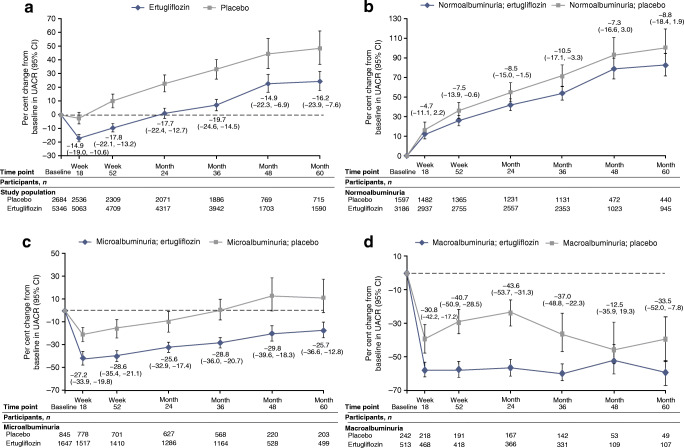
Per cent change from baseline in UACR in the overall population (**a**) and in participants with normoalbuminuria (**b**), microalbuminuria (**c**) or macroalbuminuria (**d**) at baseline (all FAS). The numbers by each time point are the placebo-adjusted difference, reported as % (95% CI). The number for treatment groups at each time point was the number of participants on study medication or who discontinued study medication ≤2 days before the sample was collected

In the overall cohort, ertugliflozin was associated with a reduction in UACR compared with placebo, and this persisted for the duration of the study (Fig. 3a). Placebo-adjusted per cent change (95% CI) in UACR at 18 weeks was −14.9 (−19.0, −10.6) in the ertugliflozin group. At month 60, the placebo-adjusted per cent change (95% CI) in UACR in the overall cohort was −16.2 (−23.9, −7.6) in the ertugliflozin group. Results by ertugliflozin dose were similar to those for the pooled cohort; at month 60, the placebo-adjusted per cent change (95% CI) in UACR was −8.3 (−17.9, 2.5) and −23.5 (−31.5, −14.5) in the ertugliflozin 5 mg and ertugliflozin 15 mg groups, respectively (ESM Fig. 4). In subgroups defined by baseline kidney function, placebo-corrected changes from baseline with ertugliflozin were larger in the subgroups of participants who had elevated albuminuria at baseline (Fig. 3b–d and ESM Figs 5 and 6). At month 60, placebo-adjusted per cent changes from baseline in UACR (95% CI) were −8.8 (−18.4, 1.9), −25.7 (−36.6, −12.8) and −33.5 (−52.0, −7.8) in participants treated with ertugliflozin with baseline normoalbuminuria, microalbuminuria and macroalbuminuria, respectively.

### eGFR change over time

**Fig. 4:**
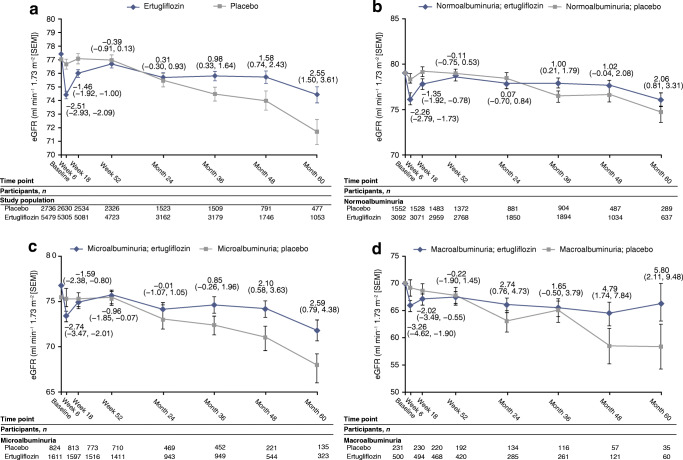
eGFR over time in the overall population (**a**) and in participants with normoalbuminuria (**b**), microalbuminuria (**c**) or macroalbuminuria (**d**) at baseline (all FAS). The numbers by each time point are the placebo-adjusted difference, reported as least squares mean (95% CI). eGFR was calculated using the CKD-EPI equation. The number for treatment group at each time point was the number of participants on study medication or who discontinued study medication ≤2 days before the sample was collected

In the overall cohort, least squares mean change (95% CI) in eGFR from baseline to week 6 was −3.0 ml min^−1^ [1.73 m]^−2^ (−3.2, −2.7) and −0.5 ml min^−1^ [1.73 m]^−2^ (−0.8, −0.1) in the ertugliflozin and placebo groups, respectively (Fig. 4, calculated using the CKD-EPI equation). After week 6, ertugliflozin was associated with an attenuation in the decline of eGFR over time compared with placebo; at month 60, the difference in least squares mean change (95% CI) in eGFR from baseline with ertugliflozin compared with placebo was 2.55 ml min^−1^ [1.73 m]^−2^ (1.50, 3.61) (*p* < 0.01). Values of eGFR over time using the MDRD equation were similar when compared with calculations using the CKD-EPI equation (ESM Fig. 7a). Changes in eGFR over time by ertugliflozin dose when calculated by the CKD-EPI and MDRD equations were similar to those for the pooled ertugliflozin population (ESM Fig. 7b,c). Ertugliflozin was associated with consistent attenuation of eGFR decline across subgroups, with a suggested larger effect observed in the macroalbuminuria and KDIGO CKD high-/very high-risk subgroups (Fig. 4b–d and ESM Figs 8, 9). At month 60, placebo-adjusted change from baseline in eGFR (ml min^−1^ [1.73 m]^−2^ [95% CI]) was 2.06 (0.81, 3.31), 2.59 (0.79, 4.38) and 5.80 (2.11, 9.48) in participants treated with ertugliflozin who had normoalbuminuria, microalbuminuria and macroalbuminuria at baseline, respectively.

### Risk for acute renal failure

In the overall population, the proportion of participants with one or more kidney-related adverse events (utilising the SMQ) did not differ between the ertugliflozin and placebo groups (4.2% and 4.7%, respectively). The event rates per 1000 person-years were 14.5 (ertugliflozin group) and 16.9 (placebo group), with a HR (95% CI) of 0.87 (0.70, 1.07). The most frequent preferred term from the SMQ was acute kidney injury (AKI), the frequency of which was 2.2% and 1.8% for the placebo and ertugliflozin groups, respectively. These kidney-related safety findings were similar in participants with baseline eGFR <60 and ≥60 ml min^−1^ [1.73 m]^−2^. The proportion of participants with a baseline eGFR <60 ml min^−1^ [1.73 m]^−2^ with one or more kidney-related adverse events was similar in the ertugliflozin and placebo groups (both 10.2%). In the subgroup with baseline eGFR ≥60 ml min^−1^ [1.73 m]^−2^, the proportion of participants with one or more kidney-related adverse events was similar in the ertugliflozin and placebo groups (2.5% and 3.1%, respectively).

## Discussion

VERTIS CV is the fourth SGLT2 inhibitor CVOT to evaluate kidney outcomes [7–9]. These outcomes have also been examined in the dedicated DKD trial, Canagliflozin and Renal Events in Diabetes with Established Nephropathy Clinical Evaluation (CREDENCE) [10]. The results of the VERTIS CV trial provide further evidence supporting the beneficial effects of this drug class on cardiovascular and kidney outcomes. Based on this body of evidence, these therapies have led to major revisions of clinical practice guidelines for type 2 diabetes mellitus [16]. Our major findings for ertugliflozin were as follows: reduced risk of the pre-specified exploratory renal composite, which included a sustained 40% decline in eGFR, chronic renal replacement therapy or renal death; significantly reduced UACR compared with placebo in participants with microalbuminuria or macroalbuminuria at baseline; preserved kidney function, especially in participants with macroalbuminuria at greatest risk of DKD progression; and a renal safety profile that was consistent with the known effects of SGLT2 inhibitors.

In previous CVOTs, SGLT2 inhibition reduced the risk of important kidney secondary endpoints. In the Empagliflozin Cardiovascular Outcome Event Trial in Type 2 Diabetes Mellitus Patients–Removing Excess Glucose (EMPA-REG OUTCOME), empagliflozin reduced the risk of the composite kidney endpoint that included doubling of serum creatinine as a measure of substantial decline in kidney function by 46%. In the Canagliflozin Cardiovascular Assessment Study (CANVAS) Program, involving a predominant atherosclerotic CVD cohort, canagliflozin reduced the risk of sustained 40% decline in eGFR, kidney replacement therapy or renal death by 40% [7, 8]. Interestingly, even in the lowest risk cohort for atherosclerotic CVD and kidney disease progression enrolled in the Dapagliflozin Effect on Cardiovascular Events–Thrombolysis in Myocardial Infarction 58 (DECLARE-TIMI 58) study, the renal composite was reduced significantly with dapagliflozin vs placebo when significant kidney function decline was defined by a sustained 40% decline in eGFR to <60 ml min^−1^ [1.73 m]^−2^ [9]. In the VERTIS CV study, the secondary outcome of a kidney composite involving a doubling of creatinine did not reach statistical significance [12]. A possible explanation for the difference is that these four studies had inconsistent definitions of the kidney composite, specifically regarding the use of doubling of serum creatinine (without a concomitant eGFR level below 45 ml min^−1^ [1.73 m]^−2^, as was used in EMPA-REG OUTCOME) and the requirement for sustainability of the decline. Consistent with previous CVOTs [17, 18], ertugliflozin reduced the risk of the renal composite endpoint by 34% when sustained 40% decline in eGFR was used instead of doubling of serum creatinine. The reduction in RR for the kidney composite was observed across CKD stages, levels of UACR and KDIGO CKD risk categories. Although the definition of significant renal function decline using sustained doubling of creatinine is considered accurate as a renal endpoint, the overall risk of DKD progression in this cohort was low and hence the power to demonstrate kidney function loss of this magnitude was insufficient. By contrast, kidney function preservation using the definition of a sustained 40% decline in eGFR is likely a more precise measure, especially in cohorts at lower risk for DKD progression, and therefore more appropriate in this setting.

The effect of SGLT2 inhibitors on UACR lowering has been consistently demonstrated, occurring in individuals with type 2 diabetes with preserved and impaired kidney function [6, 19], and tends to appear quickly after initiation of therapy, regardless of background therapy [6, 20]. Therefore, the impact of ertugliflozin on UACR in individuals with elevated UACR at baseline was consistent with previous literature. The observations that ertugliflozin reduced albuminuria progression and increased albuminuria regression are also aligned with known effects of SGLT2 inhibitors [6]. An expanding body of evidence supports the hypothesis that reduced intraglomerular pressure on the basis of natriuresis-activated tubuloglomerular feedback and other vasoactive pathways attenuates glomerular hypertension [21, 22]. These haemodynamic mechanisms are perhaps best illustrated by the observations that UACR lowering occurs rapidly and is reversible shortly after cessation of therapy [6], and by the fact that markers of reduced glomerular hypertension have been shown consistently in response to SGLT2 inhibitors [21–23]. SGLT2 inhibitor trials have also served to identify patient clinical profiles linked with kidney protection with these agents, especially people with albuminuria, as demonstrated by the rapid rate of eGFR decline in patients with macroalbuminuria treated with placebo, emphasising the importance of measuring UACR according to established clinical practice guidelines [17, 18].

SGLT2 inhibitors have characteristic effects on kidney function. In human mechanistic studies, empagliflozin reduced inulin-based GFR and renal blood flow in individuals with type 1 diabetes [21], in conjunction with an increase in urine adenosine excretion [24] and a rise in renal vascular resistance and afferent constriction [25], consistent with activation of tubuloglomerular feedback [26]. Effects on eGFR occur acutely after a single dose [27] and then persist over time [8]. Following the cessation of SGLT2 inhibitor therapy, eGFR rebounds rapidly back towards baseline [8], consistent with the haemodynamic effects of these agents [6, 28]. In the current analysis, eGFR declined acutely with ertugliflozin and was then better preserved over time compared with placebo. Consistent with results of other SGLT2 inhibitor trials, a larger effect of eGFR preservation over time (as measured by placebo-adjusted difference) was observed in the subgroup of participants who had macroalbuminuria at baseline (Fig. 3d) [6].

Reports from the US Food and Drug Administration’s adverse event reporting system suggested higher AKI risk with SGLT2 inhibitors. In individual CVOTs, AKI risk was either neutral or reduced with SGLT2 inhibitors, and in meta-analyses involving cardiorenal outcome studies, AKI risk was reduced by approximately 25% [7–10, 17, 18]. Similarly, in propensity-matched score analyses with electronic medical record data, AKI risk was lower with SGLT2 inhibitors than with other glucose-lowering therapies [29]. Consistent with this previous literature, our analysis found no indication of an increase in AKI risk with ertugliflozin.

Our analysis does have limitations. We recognise that although observations from this analysis are generally consistent with renal-related effects in other trials with SGLT2 inhibitors, findings from this cohort with type 2 diabetes and established atherosclerotic CVD may not be generalisable to other groups of individuals. We used pre-specified exploratory endpoints and did not control for type 1 error. The protocol amendment at the end of 2015 resulted in two cohorts with different durations of follow-up. A subgroup analysis of the interaction of the secondary kidney outcome by cohort was found to be non-significant. As reported in previous event-driven trials, the number of participants decreased through the observation period, limiting the interpretability of the results for eGFR and UACR over time. Although valuable as a measure of long-term kidney risk, UACR does have inherent limitations due, in part, to variability of single UACR measures. Nevertheless, the VERTIS CV trial included a large sample size, which mitigated some of the inherent limitations around the use of single UACR measurements at each time point. Furthermore, the use of single measurements and related variability would have biased our analysis towards null. Hence, the effects of ertugliflozin may be underestimated. Early and late haemodynamic effects of the SGLT2 inhibitor class of medications may limit the interpretability of the UACR results. The assessment of acute renal failure was based on adverse event reporting by investigators; however, a blinded external independent panel adjudicated all important renal events for assessment of causality with study medication. Finally, we recognise that the endpoints were exploratory in nature; however, the use of sustained 40% decline in eGFR rather than doubling of serum creatinine is widely recognised as being clinically relevant and has been used in other kidney protection studies [7, 9, 15, 30, 31].

In conclusion, when used in addition to standard of care medications, ertugliflozin was associated with a decrease in the risk of a sustained 40% decline in eGFR, with less albuminuria and with preservation of eGFR over time in individuals with type 2 diabetes and established atherosclerotic CVD. Observations from the VERTIS CV study suggest that the effects of ertugliflozin on the kidneys are generally consistent with the known benefits of SGLT2 inhibitors.

## Supplementary Information

ESM Statistical Analysis Plan (PDF 399 kb)

ESM Tables and Figures (PDF 899 kb)

## Data Availability

The data sharing policy, including restrictions, of Merck Sharp & Dohme Corp., a subsidiary of Merck & Co., Inc. (Kenilworth, NJ, USA), is available at http://engagezone.msd.com/ds_documentation.php. Requests for access to the clinical study data can be submitted through the EngageZone site or via email to dataaccess@merck.com.

## Abbreviations

AKI: Acute kidney injury
CKD: Chronic kidney disease
CKD-EPI: Chronic Kidney Disease Epidemiology Collaboration
cLDA: Constrained longitudinal data analysis
CVOT: Cardiovascular outcomes trial
DKD: Diabetic kidney disease
FAS: Full analysis set
KDIGO CKD: Kidney Disease: Improving Global Outcomes in Chronic Kidney Disease
MDRD: Modification of Diet in Renal Disease
RMANCOVA: Repeated measures ANCOVA
SBP: Systolic BP
SGLT2: Sodium–glucose cotransporter 2
SMQ: Standard Medical Dictionary for Regulatory Activities Query
UACR: Urine albumin/creatinine ratio
VERTIS CV: eValuation of ERTugliflozin effIcacy and Safety CardioVascular outcomes

## References

[1] Liu J, Tarasenko L, Terra SG (2019). Efficacy of ertugliflozin in monotherapy or combination therapy in patients with type 2 diabetes: A pooled analysis of placebo-controlled studies. Diab Vasc Dis Res.

[2] Lytvyn Y, Bjornstad P, Udell JA, Lovshin JA, Cherney DZI (2017). Sodium glucose cotransporter-2 inhibition in heart failure: Potential mechanisms, clinical applications, and summary of clinical trials. Circulation.

[3] Grunberger G, Camp S, Johnson J (2018). Ertugliflozin in patients with stage 3 chronic kidney disease and type 2 diabetes mellitus: The VERTIS RENAL randomized study. Diabetes Ther.

[4] Sahasrabudhe V, Terra SG, Hickman A (2017). The effect of renal impairment on the pharmacokinetics and pharmacodynamics of ertugliflozin in subjects with type 2 diabetes mellitus. J Clin Pharmacol.

[5] Cherney DZI, Cooper ME, Tikkanen I (2017). Pooled analysis of phase III trials indicate contrasting influences of renal function on blood pressure, body weight, and HbA1c reductions with empagliflozin. Kidney Int.

[6] Cherney DZI, Zinman B, Inzucchi SE (2017). Effects of empagliflozin on the urinary albumin-to-creatinine ratio in patients with type 2 diabetes and established cardiovascular disease: An exploratory analysis from the EMPA-REG OUTCOME randomised, placebo-controlled trial. Lancet Diabetes Endocrinol.

[7] Neal B, Perkovic V, Mahaffey KW (2017). Canagliflozin and cardiovascular and renal events in type 2 diabetes. N Engl J Med.

[8] Wanner C, Inzucchi SE, Lachin JM (2016). Empagliflozin and progression of kidney disease in type 2 diabetes. N Engl J Med.

[9] Wiviott SD, Raz I, Bonaca MP (2019). Dapagliflozin and cardiovascular outcomes in type 2 diabetes. N Engl J Med.

[10] Perkovic V, Jardine MJ, Neal B (2019). Canagliflozin and renal outcomes in type 2 diabetes and nephropathy. N Engl J Med.

[11] Cannon CP, McGuire DK, Pratley R (2018). Design and baseline characteristics of the eValuation of ERTugliflozin effIcacy and Safety CardioVascular outcomes trial (VERTIS-CV). Am Heart J.

[12] Cannon CP, Pratley R, Dagogo-Jack S (2020). Cardiovascular outcomes with ertugliflozin in type 2 diabetes. N Engl J Med.

[13] Levey AS, Coresh J, Greene T (2006). Using standardized serum creatinine values in the modification of diet in renal disease study equation for estimating glomerular filtration rate. Ann Intern Med.

[14] Work Group of Kidney Disease: Improving Global Outcomes (KDIGO) (2013). Chapter 1: Definition and classification of CKD. Kidney International Supplements 2013.

[15] Levey AS, Inker LA, Matsushita K (2014). GFR decline as an end point for clinical trials in CKD: A scientific workshop sponsored by the National Kidney Foundation and the US Food and Drug Administration. Am J Kidney Dis.

[16] American Diabetes Association (2020). 11. Microvascular complications and foot care: Standards of medical care in diabetes-2020. Diabetes Care.

[17] Zelniker TA, Wiviott SD, Raz I (2019). SGLT2 inhibitors for primary and secondary prevention of cardiovascular and renal outcomes in type 2 diabetes: A systematic review and meta-analysis of cardiovascular outcome trials. Lancet.

[18] Neuen BL, Young T, Heerspink HJL (2019). SGLT2 inhibitors for the prevention of kidney failure in patients with type 2 diabetes: A systematic review and meta-analysis. Lancet Diabetes Endocrinol.

[19] Fioretto P, Stefansson BV, Johnsson E, Cain VA, Sjostrom CD (2016). Dapagliflozin reduces albuminuria over 2 years in patients with type 2 diabetes mellitus and renal impairment. Diabetologia.

[20] Cherney D, Lund SS, Perkins BA (2016). The effect of sodium glucose cotransporter 2 inhibition with empagliflozin on microalbuminuria and macroalbuminuria in patients with type 2 diabetes. Diabetologia.

[21] Cherney DZ, Perkins BA, Soleymanlou N (2014). Renal hemodynamic effect of sodium-glucose cotransporter 2 inhibition in patients with type 1 diabetes mellitus. Circulation.

[22] van Bommel EJM, Muskiet MHA, van Baar MJB (2020). The renal hemodynamic effects of the SGLT2 inhibitor dapagliflozin are caused by post-glomerular vasodilatation rather than pre-glomerular vasoconstriction in metformin-treated patients with type 2 diabetes in the randomized, double-blind RED trial. Kidney Int.

[23] Kidokoro K, Cherney DZI, Bozovic A (2019). Evaluation of glomerular hemodynamic function by empagliflozin in diabetic mice using in vivo imaging. Circulation.

[24] Rajasekeran H, Lytvyn Y, Bozovic A (2017). Urinary adenosine excretion in type 1 diabetes. Am J Physiol Renal Physiol.

[25] Skrtic M, Yang GK, Perkins BA (2014). Characterisation of glomerular haemodynamic responses to SGLT2 inhibition in patients with type 1 diabetes and renal hyperfiltration. Diabetologia.

[26] Vallon V, Thomson SC (2017). Targeting renal glucose reabsorption to treat hyperglycaemia: The pleiotropic effects of SGLT2 inhibition. Diabetologia.

[27] Bjornstad P, Laffel L, Tamborlane WV (2018). Acute effect of empagliflozin on fractional excretion of sodium and eGFR in youth with type 2 diabetes. Diabetes Care.

[28] Barnett AH, Mithal A, Manassie J (2014). Efficacy and safety of empagliflozin added to existing antidiabetes treatment in patients with type 2 diabetes and chronic kidney disease: A randomised, double-blind, placebo-controlled trial. Lancet Diabetes Endocrinol.

[29] Iskander C, Cherney DZI, Clemens KK (2020). Use of sodium–glucose cotransporter-2 inhibitors and risk of acute kidney injury in older adults with diabetes: A population-based cohort study. Can Med Assoc J.

[30] Lambers Heerspink HJ, Perkovic V, de Zeeuw D (2011). Is doubling of serum creatinine a valid clinical 'hard' endpoint in clinical nephrology trials?. Nephron Clin Pract.

[31] Coresh J, Turin TC, Matsushita K (2014). Decline in estimated glomerular filtration rate and subsequent risk of end-stage renal disease and mortality. JAMA.

