# Supplementary material for: Effects of ertugliflozin on kidney composite outcomes, renal function and albuminuria in patients with type 2 diabetes mellitus: an analysis from the randomised VERTIS CV trial
Source: Diabetologia. 2021 Mar 4;64(6):1256–67. doi: 10.1007/s00125-021-05407-5 (PMC8099851; doi:10.1007/s00125-021-05407-5)
Supplement: Supplementary file 1 — (PDF 399 kb) [file 125_2021_5407_MOESM1_ESM.pdf]

ESM Statistical Analysis Plan

Publication for Key Kidney Outcomes

VERTIS-CV - Protocol MK8835-004-01/B1521021

Table of Contents

1. INTRODUCTION 5
2. OBJECTIVES 5
3. ANALYSIS SETS 5
4. CATEGORICAL VARIABLES AND ANALYSIS ENDPOINTS 6
   4.1 DEFINITIONS OF CATEGORICAL VARIABLES 6
   4.2 ANALYSIS ENDPOINTS 7
5. STATISTICAL METHODS 7
6. HANDLING OF MISSING VALUES 8
7. GRAPHICAL SUMMARIES 8
8. SUMMARY OF ANALYSES 10
9. DERIVATION OF EGFR (CKD-EPID) 12

Revision History

Date	Version	Comments
March 24, 2020		Final version
July 13, 2020	V1	Changes for clarifications:<br><br>1. Adding statement “Data obtained more than 2 days after the last dose of study medication were excluded for the mean change from baseline of the eGFR and UACR analyses by timepoint.” on Section 3: Analysis Sets. This statement is consistent to the CSR analyses.<br><br>2. Adding statements “Serum creatinine was collected 6 times during the first year and three times per year thereafter, urine samples for the UACR were collected twice during the first year and annually thereafter. For the assessments of events related to changes in serum creatinine local laboratory results were also used”.<br><br>3. In Statistical Section 5, adding fixed term in RMANCOVA model for baseline value of response variable. This term was missed in previous version. The current version is consistent to CSR analysis.

		4. In Handling of Missing Values, Section 6, no event has occurred in time-to-event endpoints, subjects will be censored at the time of “*the last follow-up visit*” to replace “*discontinuation of study treatment*”. This was a typo in the previous version.

1. Introduction

This SAP summarizes the analysis plan for key kidney outcomes publication using data from patients treated with either ertugliflozin or placebo in the VERTIS-CV cardiovascular outcome trial, protocol MK-8835-004-01/B1521021. Note analyses that are already included in other SAP from this study will not be repeated.

2. Objectives

The objectives, which will be assessed by baseline renal function category, are:

- To assess demographic and baseline characteristics of subjects randomized to ertugliflozin as compared with placebo

- To assess the effect of treatment with ertugliflozin compared to placebo on

    - Time to first occurrence of the following events:
        - Composite renal outcome of sustained doubling from baseline in serum creatinine (2xSCr), renal dialysis/transplant, and renal death (and individual component)
        - Composite renal outcome of sustained 40% decrease from baseline in eGFR, renal dialysis/transplant, and renal death
        - Acute renal failure (ARF) narrow SMQ
        - Progression of albuminuria
        - Regression of albuminuria

    - Change from baseline of Urinary Albumin to Creatinine Ratio (UACR) over time

    - Change from baseline over time in eGFR

3. Analysis Sets

The full analysis set (FAS) defined in Section 5.1 of the Non-CV SAP will be used for the analysis of eGFR, UACR, and renal related endpoints (data collected during the treatment period).

For AE endpoints, the All Subjects as Treated (ASaT) analysis set defined in Section 5.3 of the Non-CV SAP will be used.

For analyses that use the constrained longitudinal data analysis (cLDA) model in Section 5, subjects require at least one baseline measurement or post-baseline measurement to be included in the model.

The CV intention-to-treat (CV ITT) analysis set will be used for renal composite endpoints, their individual components, and other time-to-event endpoints. This analysis set includes all randomized subjects. CV ITT will also be used to summarize baseline characteristics.

Data obtained after the initiation of glycemic rescue therapy or after bariatric surgery will be included. Data obtained more than 2 days after the last dose of study medication were excluded for the mean change from baseline of the eGFR and UACR analyses by timepoint.

4. Categorical Variables and Analysis Endpoints

4.1 Definitions of Categorical Variables

- Treatment
    1) 2 categories: ertugliflozin pooled (5 mg and 15 mg) and placebo
    2) 3 categories: ertugliflozin 5 mg, ertugliflozin 15 mg, and placebo
    3) 4 categories: ertugliflozin 5 mg, ertugliflozin 15 mg, ertugliflozin pooled (5 mg and 15 mg), and placebo

- 3 Baseline eGFR (mL/min/1.73 m2) categories:
    1) CKD stage 1: eGFR ⩾ 90 mL/min/1.73 m^2
    2) CKD stage 2: eGFR ⩾60 and <90 mL/min/1.73 m^2
    3) CKD stage 3 or lower: eGFR <60 mL/min/1.73 m^2

- 2 Baseline eGFR (mL/min/1.73 m^2) categories:
    1) eGFR ⩾60 mL/min/1.73 m^2
    2) eGFR <60 mL/min/1.73 m^2

- Baseline UACR (mg/g) category:
    1) Normoalbuminuria (Normo): <30 mg/g
    2) Microalbuminuria (Micro): ⩾ 30 mg/g and ⩽ 300 mg/g
    3) Macroalbuminuria (Macro): > 300 mg/g

- Baseline UACR (mg/g) 2 categories:
    1) Normoalbuminuria (Normo): <30 mg/g
    2) Micro+Macro albuminuria: ⩾ 30 mg/g

- KDIGO CKD risk category at baseline
    1) Low risk: eGFR⩾ 60 mL/min/1.73 m^2 and UACR < 30 mg/g
    2) Moderate risk: eGFR⩾ 60 mL/min/1.73 m^2 and UACR < 30-300 mg/g or
eGFR 45-59 mL/min/1.73 m^2 and UACR <30 mg/g
    3) High risk: eGFR⩾ 60 mL/min/1.73 m^2 and UACR > 300 mg/g or
eGFR 45-59 mL/min/1.73 m^2 and UACR >30 mg/g or

eGFR < 45 mL/min/1.73 m^2

4.2 Analysis Endpoints

- Time to first occurrence of
  - Renal composite of sustained[1,2] 2xSCr, renal dialysis/transplant or renal death (and individual components)
  - Renal composite of sustained[1] 40% decrease eGFR, renal dialysis/transplant or renal death
  - ARF (narrow SMQ)
  - Progression of nephropathy
  - Regression of nephropathy

[1] Sustained is defined as the occurrence of a value that meets the cut-off criteria (i.e., 2xSCr, or 40% decrease) which is followed, more than 30 days later, by a subsequent value that also meets the cut-off criteria. Values that meet the cut-off criteria do not need to be proximate (there can be an intervening value that does not meet the criterion).

- Change from Baseline in eGFR (mL/min/1.73m^2) with MDRD at Week 6, Week 18, Week 52 and yearly thereafter

- Change from Baseline in eGFR (mL/min/1.73m^2) with CKD-EPI at Week 6, Week 18, Week 52 and yearly thereafter

- Change from baseline in UACR (mg/g)[2] at Week 18, 52 and yearly thereafter

[2] Serum creatinine was collected 6 times during the first year and three times per year thereafter, urine samples for the UACR were collected twice during the first year and annually thereafter. For the assessments of events related to changes in serum creatinine local laboratory results were also used.

5. Statistical Methods

The time-to-event endpoints will be analyzed using a stratified Cox proportional hazards (CPH) model including treatment group as a covariate. Cohort (defined in Section 6.2 of CV SAP) will be included as a stratification factor. For baseline subgroups (baseline eGFR category, KDIGO CKD risk categories, and UACR categories) analysis, each subgroup analysis model will include terms for treatment (categorical), subgroup (categorical), and treatment by subgroup interaction in the CPH model. The point estimates and two-sided 95% confidence intervals for the hazard ratio will be calculated from the CPH model. For subgroup analysis, the overall p-value of the interaction term will be presented.

Mean changes from baseline in eGFR (MDRD), eGFR (CKD-EPI), and UACR endpoints over time will be estimated using the constrained longitudinal data analysis (cLDA) model for the

overall population. The model will contain fixed effects for treatment, time, treatment by time interaction, and baseline HbA1c, baseline systolic blood pressure. In the model, time will be treated as a categorical variable so that no restriction will be imposed on the trajectory of the means over time. The treatment difference in terms of mean change from baseline to a given time point will be estimated and presented. An unstructured covariance matrix will be used to model the correlation among repeated measurements. If covariance structure UN does not converge, TOEP will be used. If data do not converge due to the less samples in the later timepoints (e.g., greater than 2 years), then the later points will not be included in the model and be presented by descriptive statistics.

Due to the non-normal distribution of UACR, log transformation of UACR data will be performed. The geometric means will be provided by timepoint in the data summary. Adjusted mean percentage change (derived from exponentiation of adjusted estimates) with 95% confidence intervals will be presented for each treatment. The difference between ertugliflozin treatment and placebo in mean percentage change in UACR from baseline will also be estimated.

For the subgroups: 3 eGFR categories, KDIGO CKD risk categories, and UACR categories, a repeated measures ANCOVA (RMANCOVA) method will be used. The RMANCOVA model will adjust for baseline value of response variable, baseline of HbA1c, treatment, subgroup, and treatment-by-subgroup interaction, and treatment-by-subgroup-by-time interaction. Time is treated as a categorical variable. An unstructured covariance matrix will be used to model the correlation among repeated measurements. For subgroup analyses based on factors that are already in the main model, the respective term will appear in the model only once.

Baseline demographics and disease characteristics will be summarized with the descriptive statistics by treatment (ertugliflozin 5 mg, 15 mg, pooled ertugliflozin, and placebo) for the overall population and by the following subgroups: 2 eGFR categories, 3 eGFR categories, KDIGO CKD risk categories, and UACR categories.

Section 8 contains a table which summarizes the overall analysis plan and compares to the SAP of study protocol.

6. Handling of Missing Values

No missing imputation of missing safety endpoints will be done. For analysis of UACR and eGFR, endpoints, the cLDA method uses a full likelihood model that does not require imputation of missing data and produces unbiased inference under the missingness mechanisms MCAR (missing completely at random) and MAR (missing at random). For time-to-event endpoints, if no event has occurred, subjects will be censored at the time of the last follow-up visit.

7. Graphical Summaries

Graphical summaries will include Kaplan-Meier curves of the time-to-event for each treatment group (pooled ertugliflozin doses vs. placebo) or separately by dose (ertugliflozin 5 mg, ertugliflozin 15 mg, and placebo) for the overall population and for subgroups.

The least squared mean (LSM) of change from baseline in eGFR (MDRD), eGFR (CKD-EPI) over time will be presented by treatment group for the overall population and for the following subgroups: 3 eGFR categories, UACR categories, and KDIGO CKD risk categories individually.

Additionally, adjusted mean percentage change (derived from exponentiation of adjusted estimates) in UACR over time will be presented by treatment group for the overall population and for the following subgroups: 3 eGFR categories, UACR categories, and KDIGO CKD risk categories individually.

8. Summary of Analyses

Key Kidney Outcomes					
Analysis Endpoints	Analysis Set	Statistical Method	Renal Function Category	Treatment* Group	Comparisons to SAP in the study protocol
Baseline Characteristics	CV ITT	Descriptive	• 2 eGFR categories<br>• 3 eGFR categories<br>• KDIGO CKD risk categories<br>• UACR categories	4 categories	New analyses
Time to 1st occurrence on renal composite endpoint (sustained 2xSCr, renal death, renal dialysis/transplantation) and individual component	CV ITT	CPH and KM	• Overall population<br>• 3 eGFR categories<br>• KDIGO CKD risk categories<br>• UACR 3 and 2 categories	2 categories (individual component and overall population and subgroup) and 3 categories (only for overall population)	• CV-SAP includes the overall population<br>• New analyses by baseline renal subgroup
Time to 1st occurrence on renal composite endpoint (sustained 40% decrease in eGFR, renal death, renal dialysis/transplantation) and individual component	CV ITT	CPH and KM	• Overall population<br>• 3 eGFR categories<br>• KDIGO CKD risk categories<br>• UACR 3 and 2 categories	2 categories (individual component and overall population and subgroup) and 3 categories (only for overall population)	New analyses
Change from baseline on UACR at Week 18, 52 and yearly thereafter till Year-5	FAS	cLDA RMANCOVA	• Overall population<br>• KDIGO CKD risk categories<br>• UACR categories<br>• 3 eGFR categories	2 categories and 3 categories	• Non-CV SAP includes the mean change from baseline in UACR (original scale) for the overall population<br>• New analyses using the log transformation of UACR for the overall population and by baseline renal subgroup
Time to 1st occurrence of Progression/Regression of albuminuria	CV ITT	CPH and KM	• KDIGO CKD risk categories<br>• UACR categories	2 categories and 3 categories	New analyses

Key Kidney Outcomes					
Analysis Endpoints	Analysis Set	Statistical Method	Renal Function Category	Treatment* Group	Comparisons to SAP in the study protocol
Change from baseline on eGFR (MDRD and CKD EPI) at Week 6, Week 18, Week 52 and yearly thereafter till Year-5	FAS	cLDA RMANCOVA	• Overall population<br>• 3 eGFR categories<br>• KDIGO CKD risk categories<br>• UACR categories	2 categories and 3 categories	• Non-CV SAP includes the overall population<br>• These analyses include Week 6 time point for overall population and by renal subgroups<br>• New analyses by renal subgroup<br>• New eGFR analysis calculated by CKD EPI formula
Time to 1st occurrence of Acute Renal Failure	FAS	CPH and KM	• Overall population<br>• 2 eGFR categories (<60 & ≥ 60 mL/min/1.73 m^2)	2 categories	• New analyses

[1]: 2 categories: ertugliflozin pooled (5 mg and 15 mg) and placebo
3 categories: ertugliflozin 5 mg, ertugliflozin 15 mg, and placebo
4 categories: ertugliflozin 5 mg, ertugliflozin 15 mg, ertugliflozin pooled (5 mg and 15 mg), and placebo

9. Derivation of eGFR (CKD-EPID)

The CKD-EPI equation, expressed as a single equation, is

$$GFR = 141 \times \min(Scr/\kappa, 1)^{\alpha} \times \max(Scr/\kappa, 1)^{-1.209} \times 0.993^{Age} \times 1.018 \text{ [if female]} \times 1.159 \text{ [if black]}$$

where Scr is serum creatinine, κ is 0.7 for females and 0.9 for males, α is -0.329 for females and -0.411 for males, min indicates the minimum of Scr/κor 1, and max indicates the maximum of Scr/κ or 1.

Race and Sex	Serum Creatinine µmol/L (mg/dL)	Equation
Black		
Female	≤62 (≤0.7)	$GFR = 166 \times (Scr/0.7)^{-0.329} \times (0.993)^{Age}$
	>62 (>0.7)	$GFR = 166 \times (Scr/0.7)^{-1.209} \times (0.993)^{Age}$
Male	≤80 (≤0.9)	$GFR = 163 \times (Scr/0.9)^{-0.411} \times (0.993)^{Age}$
	>80 (>0.9)	$GFR = 163 \times (Scr/0.9)^{-1.209} \times (0.993)^{Age}$
White or other		
Female	≤62 (≤0.7)	$GFR = 144 \times (Scr/0.7)^{-0.329} \times (0.993)^{Age}$
	>62 (>0.7)	$GFR = 144 \times (Scr/0.7)^{-1.209} \times (0.993)^{Age}$
Male	≤80 (≤0.9)	$GFR = 141 \times (Scr/0.9)^{-0.411} \times (0.993)^{Age}$
	>80 (>0.9)	$GFR = 141 \times (Scr/0.9)^{-1.209} \times (0.993)^{Age}$
