# Supplementary material for: Effects of ertugliflozin on kidney composite outcomes, renal function and albuminuria in patients with type 2 diabetes mellitus: an analysis from the randomised VERTIS CV trial
Source: Diabetologia. 2021 Mar 4;64(6):1256–67. doi: 10.1007/s00125-021-05407-5 (PMC8099851; doi:10.1007/s00125-021-05407-5)
Supplement: Supplementary file 2 — (PDF 899 kb) [file 125_2021_5407_MOESM2_ESM.pdf]

Electronic Supplementary Materials

Supplement to: **Effects of ertugliflozin on kidney composite outcomes, renal function and albuminuria in patients with type 2 diabetes mellitus: an analysis from the randomised VERTIS CV trial**

David Z. I. Cherney[1]· Bernard Charbonnel[2]· Francesco Cosentino[3]· Samuel Dagogo-Jack[4]· Darren K. McGuire[5, 6]· Richard Pratley[7]· Weichung J. Shih[8, 9]· Robert Frederich[10]· Mario Maldonado[11]· Annpey Pong[12]· Christopher P. Cannon[13], on behalf of the VERTIS CV Investigators

[1] University of Toronto, Toronto, ON, Canada

[2] University of Nantes, Nantes, France

[3] Unit of Cardiology, Karolinska Institute & Karolinska University Hospital, Stockholm, Sweden

[4] University of Tennessee Health Science Center, Memphis, TN, USA

[5] University of Texas Southwestern Medical Center, Dallas, TX, USA

[6] Parkland Health and Hospital System, Dallas, TX, USA

[7] AdventHealth Translational Research Institute, Orlando, FL, USA

[8] Rutgers School of Public Health, New Brunswick, NJ, USA

[9] Rutgers Cancer Institute of New Jersey, New Brunswick, NJ, USA

[10] Pfizer Inc., Collegeville, PA, USA

[11] MSD Limited, London, UK

[12] Merck & Co., Inc., Kenilworth, NJ, USA

[13] Cardiovascular Division, Brigham and Women's Hospital, Harvard Medical School, Boston, MA, USA

Electronic supplementary material

Table 1 Baseline demographic and disease characteristics

Table 2 Baseline demographic and disease characteristics by baseline eGFR category

Table 3 Baseline demographic and disease characteristics by baseline UACR category

Table 4 Baseline demographic and disease characteristics by baseline KDIGO CKD risk category

Table 5 Composite of doubling of serum creatinine, kidney dialysis/transplant, or renal death by baseline kidney function categories

Table 6 Composite of sustained doubling of serum creatinine, chronic kidney dialysis/transplant, or renal death by baseline kidney function categories

Table 7 Composite of sustained 40% reduction from baseline in eGFR, chronic kidney dialysis/transplant, or by baseline kidney function categories

Table 8 Hazard models for time to first progression and regression of albuminuria status by baseline UACR and KDIGO CKD risk categories (ITT population)

Fig. 1 Prognosis of CKD by GFR and albuminuria category

Fig. 2 Kaplan–Meier plot for the time to first event in the sensitivity analysis (sustained doubling of serum creatinine, chronic kidney dialysis/transplant or renal death) (ITT)

Fig. 3 Kaplan–Meier plot for the time to first event in the pre-specified exploratory kidney composite outcome (sustained 40% decrease from baseline in eGFR, chronic renal dialysis/transplant, or renal death) by ertugliflozin dose (ITT)

Fig. 4 Per cent change from baseline in UACR by ertugliflozin dose (FAS population)

Fig. 5 Per cent change from baseline in UACR in participants assigned to the KDIGO CKD low-risk category (**a**), moderate-risk category (**b**) and high-/very high-risk category (**c**) (all FAS)

Fig. 6 Per cent change from baseline in UACR in participants with CKD stage 1 at baseline (**a**), CKD stage 2 at baseline (**b**) and CKD stage 3 at baseline (**c**) (all FAS)

Fig. 7 Mean eGFR over time in the overall population using the MDRD equation (**a**), by ertugliflozin dose using the CKD-EPI equation (**b**) and by ertugliflozin dose using the MDRD equation (**c**) (all FAS)

Fig. 8 Mean eGFR over time in participants assigned to the KDIGO CKD low-risk category (**a**), moderate-risk category (**b**) and high-/very high-risk category (**c**) (all FAS)

Fig. 9 Mean eGFR over time in participants with CKD stage 1 at baseline (**a**), CKD stage 2 at baseline (**b**) and CKD stage 3 at baseline (**c**) (all FAS)

Table 1 Baseline demographic and disease characteristics of the overall population

Characteristic	Placebo (*n*=2747)	Ertugliflozin 5 mg (*n*=2752)	Ertugliflozin 15 mg (*n*=2747)	Ertugliflozin (pooled) (*n*=5499)
Female sex, *n* (%)	844 (30.7)	801 (29.1)	832 (30.3)	1633 (29.7)
Age, years	64.4 (8.0)	64.3 (8.2)	64.4 (8.0)	64.4 (8.1)
Age, *n* (%)				
<60 years old (%)	721 (26.2)	753 (27.4)	733 (26.7)	1486 (27.0)
≥60 years old (%)	2026 (73.8)	1999 (72.6)	2014 (73.3)	4013 (73.0)
Race, *n* (%)				
White	2414 (87.9)	2390 (86.8)	2436 (88.7)	4826 (87.8)
Black or African American	69 (2.5)	91 (3.3)	75 (2.7)	166 (3.0)
Asian	162 (5.9)	187 (6.8)	149 (5.4)	336 (6.1)
Multiple	70 (2.5)	67 (2.4)	69 (2.5)	136 (2.5)
Other (American Indian, Alaska Native, Native Hawaiian or Other Pacific Islander)	32 (1.2)	17 (0.6)	18 (0.7)	35 (0.6)
HbA1c, mmol/mol	66.3 (10.3)	66.8 (10.6)	66.5 (10.4)	66.6 (10.5)
HbA1c, %	8.2 (0.9)	8.3 (1.0)	8.2 (1.0)	8.2 (1.0)
Duration of T2DM, years	13.1 (8.4)	13.1 (8.4)	12.7 (8.2)	12.9 (8.3)
Haemoglobin, g/L	139.5 (13.7)	140.3 (13.8)	139.7 (13.3)	140.0 (13.5)
Body weight, kg	91.9 (18.3)	91.9 (18.4)	91.6 (18.6)	91.7 (18.5)
BMI, kg/m^2	32.0 (5.5)	31.8 (5.3)	32.0 (5.4)	31.9 (5.4)
BMI, *n* (%)				
<30 kg/m^2	1046 (38.1)	1066 (38.7)	1066 (38.8)	2132 (38.8)
30 to <35 kg/m^2	1009 (36.7)	1016 (36.9)	979 (35.6)	1995 (36.3)
≥35 kg/m^2	692 (25.2)	668 (24.3)	701 (25.5)	1369 (24.9)
eGFR, ml min^{-1} [1.73 m]$^{-2}$ (MDRD)	75.7 (20.8)	76.0 (20.8)	76.2 (20.9)	76.1 (20.9)

UACR, mg/mmol (median [IQR])	2.1 (0.7–7.5)	2.0 (0.7–7.7)	2.1 (0.7–7.8)	2.0 (0.7–7.8)
UACR, mg/g (median [IQR])	19.0 (6.0–66.5)	18.0 (6.0–68.0)	19.0 (6.0–69.0)	18.0 (6.0–69.0)
SBP, mmHg	133.1 (13.9)	133.7 (13.7)	133.2 (13.8)	133.5 (13.7)
DBP, mmHg	76.4 (8.7)	76.8 (8.5)	76.7 (8.2)	76.8 (8.3)
LDL cholesterol, mmol/L	2.3 (1.0)	2.3 (1.0)	2.3 (1.0)	2.3 (1.0)
Glucose-lowering agents, *n* (%)				
Insulin	1344 (48.9)	1308 (47.5)	1248 (45.4)	2556 (46.5)
Biguanides	2124 (77.3)	2071 (75.3)	2097 (76.3)	4168 (75.8)
Sulphonylureas	1122 (40.8)	1121 (40.7)	1147 (41.8)	2268 (41.2)
DPP4 inhibitors	292 (10.6)	301 (10.9)	318 (11.6)	619 (11.3)
GLP-1 RA	86 (3.1)	109 (4.0)	83 (3.0)	192 (3.5)
Other	122 (4.4)	108 (3.9)	99 (3.6)	207 (3.8)
Antihypertensive agents, *n* (%)				
Antihypertensives (any)	2632 (95.8)	2608 (94.8)	2613 (95.1)	5221 (94.9)
RAAS inhibitors	2239 (81.5)	2228 (81.0)	2219 (80.8)	4447 (80.9)
Calcium channel blockers	950 (34.6)	935 (34.0)	912 (33.2)	1847 (33.6)
β-blockers	1903 (69.3)	1884 (68.5)	1905 (69.3)	3789 (68.9)
Diuretics	1196 (43.5)	1167 (42.4)	1179 (42.9)	2346 (42.7)
Antiplatelet or antithrombotic drugs, *n* (%)	2446 (89.0)	2439 (88.6)	2441 (88.9)	4880 (88.7)
Lipid-lowering agents, *n* (%)				
Statin	2242 (81.6)	2259 (82.1)	2246 (81.8)	4505 (81.9)
Ezetimibe	115 (4.2)	86 (3.1)	92 (3.3)	178 (3.2)
Fibrates	243 (8.8)	226 (8.2)	198 (7.2)	424 (7.7)

Values are mean (standard deviation) unless otherwise stated. BMI, body mass index; DBP, diastolic blood pressure; DPP4, dipeptidyl peptidase 4; GLP-1 RA, glucagon-like peptide 1 receptor agonist; IQR, interquartile range; MDRD, Modification of Diet in Renal Disease; RAAS, renin–angiotensin–aldosterone system; SBP, systolic blood pressure; T2DM, type 2 diabetes mellitus; UACR, urinary albumin-to-creatinine ratio.

Table 2 Baseline demographic and disease characteristics by baseline eGFR category

	CKD stage 1 *N*=2048		CKD stage 2 *N*=4390		CKD stage 3 *N*=1807	
Variable	Placebo *n*=678	Ertugliflozin (pooled) *n*=1370	Placebo *n*=1461	Ertugliflozin (pooled) *n*=2929	Placebo *n*=608	Ertugliflozin (pooled) *n*=1199
Age, years	60.5 (7.4)	60.2 (7.9)	64.7 (7.7)	64.7 (7.5)	68.0 (7.5)	68.3 (7.6)
Female sex, *n* (%)	175 (25.8)	324 (23.6)	458 (31.3)	868 (29.6)	211 (34.7)	440 (36.7)
Duration of T2DM, years	10.9 (6.7)	10.4 (7.0)	12.7 (8.3)	13.1 (8.3)	16.4 (9.3)	15.4 (8.8)
HbA1c, mmol/mol	67.7(10.9)	67.1 (10.8)	65.5 (10.2)	66.5 (10.5)	66.5 (9.7)	66.4 (10.1)
HbA1c, %	8.3 (1.0)	8.3 (1.0)	8.1 (0.9)	8.2 (1.0)	8.2 (0.9)	8.2 (0.9)
BMI, kg/m^2	31.5 (5.3)	31.7 (5.4)	31.9 (5.4)	31.8 (5.3)	32.7 (5.7)	32.3 (5.5)
eGFR, ml min^{-1} [1.73 m]$^{-2}$ (MDRD)	103.0 (12.3)	103.3 (12.7)	74.3 (8.2)	74.5 (8.4)	48.6 (8.0)	49.1 (8.0)
UACR, mg/mmol (median [IQR])	1.9 (0.8–6.4)	2.0 (0.8–6.6)	1.9 (0.7–6.2)	1.7 (0.7–6.4)	3.5 (0.9–13.2)	3.4 (0.9–15.4)
UACR, mg/g (median [IQR])	17.0 (7.0–57.0)	18.0 (7.0–58.0)	17.0 (6.0–55.0)	15.0 (6.0–57.0)	31.0 (8.0–117.0)	30.0 (8.0–136.0)
SBP, mmHg	132.5 (13.6)	133.3 (12.9)	133.6 (13.5)	133.3 (13.7)	132.6 (15.2)	134.1 (14.8)
Haemoglobin, g/L	142.9 (12.5)	142.6 (12.4)	140.1 (13.3)	140.7 (13.3)	134.6 (14.3)	135.4 (14.2)
Background treatment, *n* (%)						
Insulin	286 (42.2)	529 (38.6)	691 (47.3)	1335 (45.6)	367 (60.4)	692 (57.7)
Biguanides	567 (83.6)	1137 (83.0)	1169 (80.0)	2291 (78.2)	388 (63.8)	739 (61.6)
Antihypertensives, *n* (%)						
Antihypertensives (any)	631 (93.1)	1251 (91.3)	1404 (96.1)	2798 (95.5)	597 (98.2)	1171 (97.7)
RAAS inhibitors	541 (79.8)	1056 (77.1)	1195 (81.8)	2390 (81.6)	503 (82.7)	1000 (83.4)
Diuretic	216 (31.9)	430 (31.4)	624 (42.7)	1252 (42.7)	356 (58.6)	664 (55.4)

Loop diuretic	49 (7.2)	118 (8.6)	193 (13.2)	405 (13.8)	184 (30.3)	303 (25.3)
Mineralocorticoid receptor antagonists	28 (4.1)	78 (5.7)	111 (7.6)	229 (7.8)	85 (14.0)	143 (11.9)
Antiplatelet or antithrombotic drugs, *n* (%)	601 (88.6)	1201 (87.7)	1295 (88.6)	2615 (89.3)	550 (90.5)	1064 (88.7)
Lipid-lowering agents, *n* (%)	561 (82.7)	1130 (82.5)	1223 (83.7)	2478 (84.6)	529 (87.0)	1046 (87.2)

Values are mean (SD) unless otherwise stated. CKD, chronic kidney disease; IQR, interquartile range; MDRD, Modification of Diet in Renal Disease; RAAS, renin–angiotensin–aldosterone system; SBP, systolic blood pressure; T2DM, type 2 diabetes mellitus; UACR, urinary albumin-to-creatinine ratio.

Table 3 Baseline demographic and disease characteristics by baseline UACR category

	Normoalbuminuria *N*=4783		Microalbuminuria *N*=2492		Macroalbuminuria *N*=755	
Variable	Placebo *n*=1597	Ertugliflozin (pooled) *n*=3186	Placebo *n*=845	Ertugliflozin (pooled) *n*=1647	Placebo *n*=242	Ertugliflozin (pooled) *n*=513
Age, years	64.2 (8.0)	64.4 (8.1)	64.9 (8.0)	64.4 (8.0)	63.2 (7.8)	64.1 (7.8)
Female sex, *n* (%)	549 (34.4)	1025 (32.2)	210 (24.9)	446 (27.1)	65 (26.9)	108 (21.1)
Duration of T2DM, years	12.4 (8.2)	12.4 (8.2)	13.8 (8.2)	13.3 (8.3)	15.5 (9.6)	14.8 (8.0)
HbA1c, mmol/mol	65.2 (10.0)	65.5 (10.3)	67.7 (10.7)	68.0 (10.5)	68.5 (10.6)	69.3 (10.9)
HbA1c, %	8.1 (0.9)	8.1 (0.9)	8.3 (1.0)	8.4 (1.0)	8.4 (1.0)	8.5 (1.0)
BMI, kg/m^2	31.7 (5.4)	31.8 (5.3)	32.0 (5.4)	31.8 (5.2)	33.0 (5.7)	32.5 (5.7)
eGFR, ml min^{-1} [1.73 m]$^{-2}$ (MDRD)	77.5 (20.2)	77.4 (19.9)	74.4 (21.2)	75.7 (21.5)	68.3 (22.2)	68.3 (22.3)
UACR, mg/mmol (median [IQR])	0.9 (0.5–1.6)	0.9 (0.5–1.6)	7.6 (4.9–13.0)	7.8 (4.9–13.8)	84.5 (48.6–163.7)	78.6 (48.4–153.6)
UACR, mg/g (median [IQR])	8.0 (4.0–14.0)	8.0 (4.0–14.0)	67.0 (43.0–115.0)	69.0 (43.0–122.0)	748.0 (430.0–1359.0)	696.0 (428.0–1359.0)
SBP, mmHg	131.3 (13.5)	131.4 (13.3)	134.9 (14.0)	135.6 (13.4)	139.1 (13.6)	140.3 (13.8)
Haemoglobin, g/L	139.5 (13.4)	140.1 (12.9)	139.8 (13.7)	140.5 (14.1)	138.8 (15.2)	138.5 (15.4)
Background treatment, *n* (%)						
Insulin	725 (45.4)	1353 (42.5)	438 (51.8)	828 (50.3)	152 (62.8)	297 (57.9)
Biguanides	1253 (78.5)	2455 (77.1)	654 (77.4)	1247 (75.7)	169 (69.8)	360 (70.2)
Antihypertensives, *n* (%)						
Antihypertensives (any)	1535 (96.1)	3012 (94.5)	804 (95.1)	1580 (95.9)	232 (95.9)	488 (95.1)
RAAS inhibitors	1306 (81.8)	2532 (79.5)	681 (80.6)	1373 (83.4)	200 (82.6)	424 (82.7)

Diuretic	681 (42.6)	1325 (41.6)	363 (43.0)	717 (43.5)	125 (51.7)	249 (48.5)
Loop diuretic	215 (13.5)	435 (13.7)	143 (16.9)	262 (15.9)	62 (25.6)	108 (21.1)
Mineralocorticoid receptor antagonists	133 (8.3)	273 (8.6)	72 (8.5)	128 (7.8)	14 (5.8)	38 (7.4)
Antiplatelet or antithrombotic drugs, *n* (%)	1424 (89.2)	2859 (89.7)	752 (89.0)	1434 (87.1)	212 (87.6)	451 (87.9)
Lipid-lowering agents, *n* (%)	1361 (85.2)	2718 (85.3)	696 (82.4)	1377 (83.6)	203 (83.9)	431 (84.0)

Values are mean (SD) unless otherwise stated. IQR, interquartile range; MDRD, Modification of Diet in Renal Disease; RAAS, renin–angiotensin–aldosterone system; SBP, systolic blood pressure; T2DM, type 2 diabetes mellitus; UACR, urinary albumin-to-creatinine ratio.

Table 4 Baseline demographic and disease characteristics by baseline KDIGO CKD risk category

	**Baseline KDIGO CKD risk category**					
	Low risk of CKD *N*=3916		Moderate risk of CKD *N*=2568		High/very high risk of CKD *N*=1548	
Variable	Placebo *n*=1307	Ertugliflozin (pooled) *n*=2609	Placebo *n*=859	Ertugliflozin (pooled) *n*=1709	Placebo *n*=517	Ertugliflozin (pooled) *n*=1031
Age, years	63.2 (7.8)	63.4 (7.9)	65.0 (7.9)	64.5 (7.9)	66.3 (8.2)	66.5 (8.1)
Female sex, *n* (%)	426 (32.6)	785 (30.1)	245 (28.5)	499 (29.2)	152 (29.4)	297 (28.8)
Duration of T2DM, years	11.5 (7.6)	11.9 (7.9)	13.8 (8.5)	13.1 (8.4)	16.1 (9.3)	15.1 (8.5)
HbA1c, mmol/mol	65.1 (10.1)	65.5 (10.4)	67.3 (10.6)	67.3 (10.5)	67.4 (10.2)	68.1 (10.6)
HbA1c, %	8.1 (0.9)	8.1 (0.9)	8.3 (1.0)	8.3 (1.0)	8.3 (0.9)	8.4 (1.0)
BMI, kg/m^2	31.5 (5.3)	31.6 (5.2)	32.0 (5.2)	32.1 (5.3)	32.8 (6.0)	32.2 (5.5)
eGFR, ml min^{-1} [1.73 m]$^{-2}$ (MDRD)	83.8 (16.4)	83.5 (16.3)	75.2 (19.6)	75.9 (20.1)	56.3 (19.9)	57.1 (20.2)
UACR, mg/mmol (median [IQR])	0.9 (0.6–1.6)	0.9 (0.5–1.6)	5.4 (3.1–10.4)	5.4 (2.8–10.2)	25.7 (5.7–78.9)	34.0 (6.7–78.6)
UACR, mg/g (median [IQR])	8.0 (5.0–14.0)	8.0 (4.0–14.0)	48.0 (27.0–92.0)	48.0 (25.0–90.0)	227.0 (50.0–698.5)	301.0 (59.0–696.0)
SBP, mmHg	131.6 (13.4)	131.6 (13.0)	133.8 (13.6)	134.2 (13.7)	135.8 (15.3)	137.4 (14.4)
Haemoglobin, g/L	140.7 (13.0)	141.0 (12.5)	139.5 (13.7)	140.7 (13.8)	136.6 (14.9)	136.6 (15.0)
Background treatment, *n* (%)						
Insulin	557 (42.6)	1046 (40.1)	436 (50.8)	821 (48.0)	320 (61.9)	614 (59.6)
Biguanides	1067 (81.6)	2081 (79.8)	672 (78.2)	1316 (77.0)	338 (65.4)	666 (64.6)
Antihypertensives, *n* (%)						
Antihypertensives (any)	1249 (95.6)	2452 (94.0)	820 (95.5)	1635 (95.7)	501 (96.9)	996 (96.6)
RAAS inhibitors	1063 (81.3)	2066 (79.2)	699 (81.4)	1401 (82.0)	425 (82.2)	866 (84.0)

Diuretic	511 (39.1)	1006 (38.6)	363 (42.3)	733 (42.9)	295 (57.1)	554 (53.7)
Loop diuretic	141 (10.8)	290 (11.1)	120 (14.0)	264 (15.4)	158 (30.6)	252 (24.4)
Mineralocorticoid receptor antagonists	92 (7.0)	191 (7.3)	70 (8.1)	142 (8.3)	57 (11.0)	107 (10.4)
Antiplatelet or antithrombotic drugs, *n* (%)	1166 (89.2)	2345 (89.9)	757 (88.1)	1491 (87.2)	464 (89.7)	909 (88.2)
Lipid-lowering agents, *n* (%)	1112 (85.1)	2211 (84.7)	705 (82.1)	1438 (84.1)	442 (85.5)	881 (85.5)

Values are mean (SD) unless otherwise stated. CKD, chronic kidney disease; IQR, interquartile range; KDIGO CKD, Kidney Disease Improving Global Outcomes chronic kidney disease; MDRD, Modification of Diet in Renal Disease; RAAS, renin–angiotensin–aldosterone system; SBP, systolic blood pressure; T2DM, type 2 diabetes mellitus; UACR, urinary albumin-to-creatinine ratio.

Table 5 Composite of doubling of baseline serum creatinine, kidney dialysis/transplant, or renal death by baseline kidney function categories (ITT population)

		Placebo *N*=2747		Ertugliflozin *N*=5499				
**Baseline kidney function category**	**Subgroups**	***n* (%)**	**Event rate per 1000 person-years[a]**	***n* (%)**	**Event rate per 1000 person-years[a]**	**AERR**	**HR (95% CI)[b]**	***p* value for interaction[c]**
Baseline eGFR category	CKD stage 1 *N*=2048	22 (3.2)	9.3	46 (3.4)	9.6	0.3	1.04 (0.63, 1.73)	0.30
	CKD stage 2 *N*=4390	53 (3.6)	10.5	71 (2.4)	7.0	−3.5	0.66 (0.46, 0.94)	
	CKD stage 3 *N*=1807	33 (5.4)	16.3	58 (4.8)	14.7	−1.6	0.90 (0.59, 1.38)	
Baseline UACR category	Normoalbuminuria *N*=4783	35 (2.2)	6.3	64 (2.0)	5.8	−0.5	0.92 (0.61, 1.39)	0.43

	Microalbuminuria<br>*N*=2492	36 (4.3)	12.6	57 (3.5)	10.0	−2.6	0.80 (0.53, 1.21)	
	Macroalbuminuria<br>*N*=755	37 (15.3)	50.0	50 (9.7)	31.7	−18.3	0.62 (0.41, 0.95)	
Baseline KDIGO CKD risk category	Low risk of CKD<br>*N*=3916	30 (2.3)	6.5	45 (1.7)	4.9	−1.6	0.75 (0.48, 1.20)	0.69
	Moderate risk of CKD<br>*N*=2568	32 (3.7)	10.9	60 (3.5)	10.1	−0.8	0.93 (0.61, 1.43)	
	High/very high risk of CKD<br>*N*=1548	46 (8.9)	27.6	67 (6.5)	20.3	−7.3	0.73 (0.50, 1.06)	

[a]Person-years is calculated as the sum of the participants´ time to first event or time to censoring (the earliest of a participants´ end of study date, death date, or last contact date).

[b]Ertugliflozin versus placebo, based on the stratified Cox proportional hazards model that includes treatment, subgroup, and treatment-by-subgroup interaction as explanatory factors and cohort category as a stratification factor.

[c]The interaction *p* value is for the treatment-by-subgroup interaction.

AERR, absolute event rate reduction; CKD, chronic kidney disease; ITT, intention to treat; KDIGO CKD, Kidney Disease Improving Global Outcomes chronic kidney disease; UACR, urinary albumin-to-creatinine ratio.

Table 6 Composite of sustained doubling of serum creatinine, chronic kidney dialysis/transplant, or renal death by baseline kidney function categories (ITT population)

		Placebo *N*=2747		Ertugliflozin *N*=5499				
**Baseline kidney function category**	**Subgroups**	***n* (%)**	**Event rate per 1000 person-years[a]**	***n* (%)**	**Event rate per 1000 person-years[a]**	**AERR**	**HR (95% CI)[b]**	***p* value for interaction[c]**
Baseline eGFR category	CKD stage 1 *N*=2048	12 (1.8)	5.0	10 (0.7)	2.1	−2.9	0.41 (0.18, 0.96)	0.45
	CKD stage 2 *N*=4390	11 (0.8)	2.2	18 (0.6)	1.8	−0.4	0.81 (0.38, 1.71)	
	CKD stage 3 *N*=1807	10 (1.6)	4.9	15 (1.3)	3.8	−1.1	0.77 (0.34, 1.71)	
Baseline UACR category	Normoalbuminuria *N*=4783	9 (0.6)	1.6	7 (0.2)	0.6	−1.0	0.39 (0.15, 1.05)	0.32

	Microalbuminuria<br>*N*=2492	7 (0.8)	2.4	15 (0.9)	2.6	0.2	1.07 (0.44, 2.62)	
	Macroalbuminuria<br>UACR >3.39 mg/mmol<br>*N*=755	17 (7.0)	22.4	21 (4.1)	13.0	−9.4	0.58 (0.31, 1.11)	
Baseline KDIGO CKD risk category	Low risk of CKD<br>*N*=3916	9 (0.7)	1.9	6 (0.2)	0.7	−1.2	0.34 (0.12, 0.95)	0.21
	Moderate risk of CKD<br>*N*=2568	5 (0.6)	1.7	13 (0.8)	2.2	0.5	1.26 (0.45, 3.53)	
	High/very high risk of CKD<br>*N*=1548	19 (3.7)	11.2	24 (2.3)	7.2	−4.0	0.64 (0.35, 1.18)	

[a]Person-years is calculated as the sum of the participants´ time to first event or time to censoring (the earliest of a participants´ end of study date, death date, or last contact date).

[b]Ertugliflozin versus placebo, based on the stratified Cox proportional hazards model that includes treatment, subgroup, and treatment-by-subgroup interaction as explanatory factors and cohort category as a stratification factor.

[c]The interaction *p* value is for the treatment-by-subgroup interaction.

AERR, absolute event rate reduction; CKD, chronic kidney disease; ITT, intention to treat; KDIGO CKD, Kidney Disease Improving Global Outcomes chronic kidney disease; UACR, urinary albumin-to-creatinine ratio.

Table 7 Composite of sustained 40% reduction from baseline in eGFR, chronic kidney dialysis/transplant, or renal death by baseline kidney function categories (ITT population)

		Placebo *N*=2747		Ertugliflozin *N*=5499				
**Baseline kidney function category**	**Subgroups**	***n* (%)**	**Event rate per 1000 person-years[a]**	***n* (%)**	**Event rate per 1000 person-years[a]**	**AERR**	**HR (95% CI)[b]**	***p* value for interaction[c]**
Baseline eGFR category	CKD stage 1 *N*=2048	22 (3.2)	9.3	39 (2.9)	8.2	−1.1	0.89 (0.53, 1.49)	0.10
	CKD stage 2 *N*=4390	44 (3.0)	8.8	42 (1.4)	4.1	−4.7	0.47 (0.31, 0.72)	
	CKD stage 3 *N*=1807	19 (3.1)	9.4	32 (2.7)	8.1	−1.3	0.86 (0.49, 1.52)	
Baseline UACR category	Normoalbuminuria *N*=4783	34 (2.1)	6.1	32 (1.0)	2.9	−3.2	0.47 (0.29, 0.77)	0.16

	Microalbuminuria *N*=2492	21 (2.5)	7.3	39 (2.4)	6.9	−0.4	0.93 (0.55, 1.59)	
	Macroalbuminuria *N*=755	30 (12.4)	40.8	36 (7.0)	22.7	−18.1	0.56 (0.35, 0.91)	
Baseline KDIGO CKD risk category	Low risk of CKD *N*=3916	31 (2.4)	6.8	27 (1.0)	3.0	−3.8	0.44 (0.26, 0.74)	0.17
	Moderate risk of CKD *N*=2568	20 (2.3)	6.8	37 (2.2)	6.2	−0.6	0.90 (0.52, 1.55)	
	High/very high risk of CKD *N*=1548	34 (6.6)	20.3	43 (4.2)	13.0	−7.3	0.64 (0.41, 1.01)	

[a]Person-years is calculated as the sum of the participants´ time to first event or time to censoring (the earliest of a participants´ end of study date, death date, or last contact date).

[b]Ertugliflozin versus placebo, based on the stratified Cox proportional hazards model that includes treatment, subgroup, and treatment-by-subgroup interaction as explanatory factors and cohort category as a stratification factor.

[c]The interaction *p* value is for the treatment-by-subgroup interaction.

AERR, absolute event rate reduction; CKD, chronic kidney disease; ITT, intention to treat; KDIGO CKD, Kidney Disease Improving Global Outcomes chronic kidney disease; UACR, urinary albumin-to-creatinine ratio.

Table 8 Hazard models for time to first progression and regression of albuminuria status by baseline UACR and KDIGO CKD risk categories (ITT population)

Baseline kidney function category	Subgroups	Placebo *N*=2747 *n* (%)	Placebo *N*=2747 Event rate per 1000 person-years[a]	Ertugliflozin *N*=5499 *n* (%)	Ertugliflozin *N*=5499 Event rate per 1000 person-years[a]	AERR	HR (95% CI)[b]	*p* value for interaction[c]
**Progression of UACR**								
Baseline UACR category	Normoalbuminuria *N*=4783	575 (36.0)	162.6	1051 (33.0)	137.4	−25.2	0.85 (0.77, 0.94)	0.02
	Microalbuminuria *N*=2492	192 (22.7)	95.1	259 (15.7)	58.8	−36.3	0.62 (0.52, 0.75)	
Baseline KDIGO CKD risk category	Low risk of CKD *N*=3916	453 (34.7)	151.2	838 (32.1)	130.1	−21.1	0.86 (0.77, 0.97)	0.05
	Moderate risk of CKD *N*=2568	232 (27.0)	116.0	352 (20.6)	78.7	−37.3	0.68 (0.58, 0.81)	

	High/very high risk of CKD<br>*N*=1548	82 (15.9)	69.9	120 (11.6)	48.2	−21.7	0.69 (0.52, 0.92)	
**Regression of UACR**								
Baseline UACR category	Microalbuminuria<br>*N*=2492	387 (45.8)	253.2	871 (52.9)	311.0	57.8	1.24 (1.10, 1.39)	0.04
	Macroalbuminuria<br>*N*=755	100 (41.3)	239.2	304 (59.3)	439.7	200.5	1.71 (1.37, 2.15)	
Baseline KDIGO CKD risk category	Moderate risk of CKD<br>*N*=2568	293 (34.1)	165.3	688 (40.3)	202.3	37.0	1.24 (1.08, 1.42)	0.61
	High/very high risk of CKD<br>*N*=1548	193 (37.3)	204.4	484 (46.9)	288.1	83.7	1.38 (1.17, 1.63)	

[a]Person-years is calculated as the sum of the participants´ time to first event or time to censoring (the earliest of a participants´ end of study date, death date, or last contact date).

[b]Ertugliflozin versus placebo, based on the stratified Cox proportional hazards model that includes treatment, subgroup, and treatment-by-subgroup interaction as explanatory factors and cohort category as a stratification factor.

[c]The interaction *p* value is for the treatment-by-subgroup interaction.

AERR, absolute event rate reduction; CKD, chronic kidney disease; ITT, intention to treat; KDIGO CKD, Kidney Disease Improving Global Outcomes chronic kidney disease; UACR, urinary albumin-to-creatinine ratio.

Fig. 1 Prognosis of CKD by GFR and albuminuria category

Prognosis of CKD by GFR and albuminuria categories: KDIGO 2012				Persistent albuminuria categories Description and range		
				A1	A2	A3
				Normal to mildly increased	Moderately increased	Severely increased
				<30 mg/g <3 mg/mmol	30–300 mg/g 3–30 mg/mmol	>300 mg/g >30 mg/mmol
GFR categories (ml min^{-1} 1.73 m^{-2}) Description and range	G1	Normal or high	≥90	Low risk	Moderate risk	High risk
	G2	Mildly decreased	60–89	Low risk	Moderate risk	High risk
	G3a	Mildly to moderately decreased	45–59	Moderate risk	High risk	Very high risk
	G3b	Moderately to severely decreased	30–44	High risk	Very high risk	Very high risk
	G4	Severely decreased	15–29	Patients with eGFR <30 ml min^{-1} 1.73 m^{-2} excluded from VERTIS CV		
	G5	Kidney failure	<15			

Participants assigned to the high- and very high-risk categories were pooled for the analyses. Green, low risk (if no other markers of kidney disease, no CKD); Yellow, moderately increased risk; Orange, high risk; Red, very high risk. CKD, chronic kidney disease; KDIGO, Kidney Disease: Improving Global Outcomes Prognosis of CKD by GFR and albuminuria category. Reprinted from Kidney international supplements, 3(1), Chapter 1: Definition and classification of CKD, 19–62, Copyright (2013), with permission from Elsevier.

Fig. 2 Kaplan–Meier plot for the time to first event in the sensitivity analysis (sustained doubling of serum creatinine, chronic kidney dialysis/transplant or renal death) (ITT)

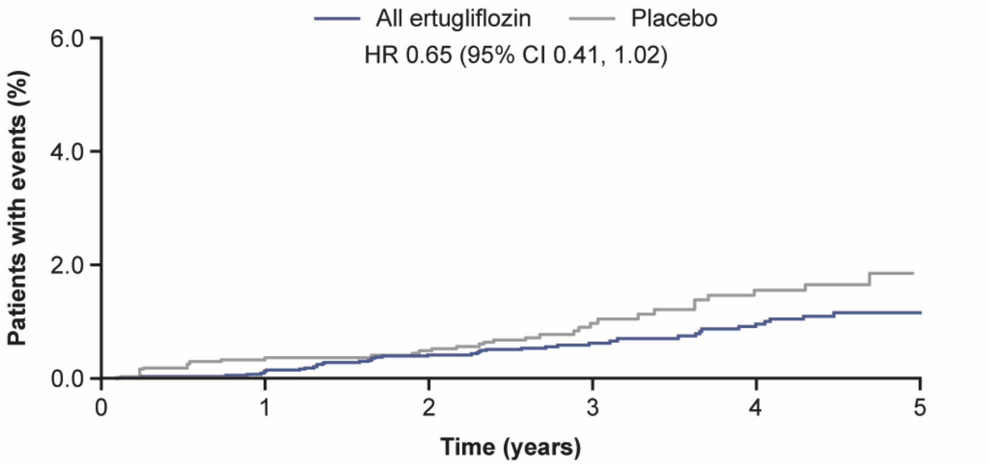

Number of patients at risk

All ertugliflozin	5499	5320	5161	2788	2318	412
Placebo	2747	2657	2575	1384	1137	221

ITT, intention to treat.

Fig. 3 Kaplan–Meier plot for the time to first event in the pre-specified exploratory kidney composite outcome (sustained 40% decrease from baseline in eGFR, chronic renal dialysis/transplant, or renal death) by ertugliflozin dose (ITT)

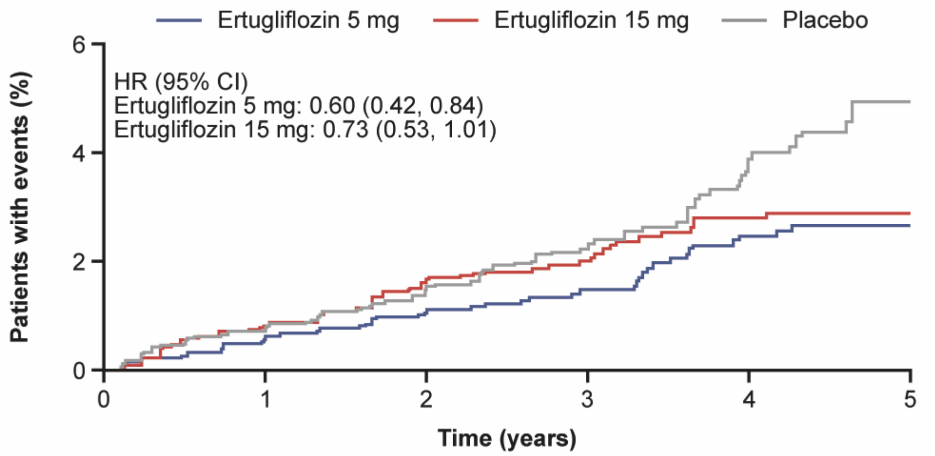

Number of patients at risk						
Ertugliflozin 5 mg	2752	2653	2575	1379	1143	201
Ertugliflozin 15 mg	2747	2635	2539	1378	1133	209
Placebo	2747	2648	2550	1366	1112	212

ITT, intention to treat.

Fig. 4 Per cent change from baseline in UACR by ertugliflozin dose (FAS population)

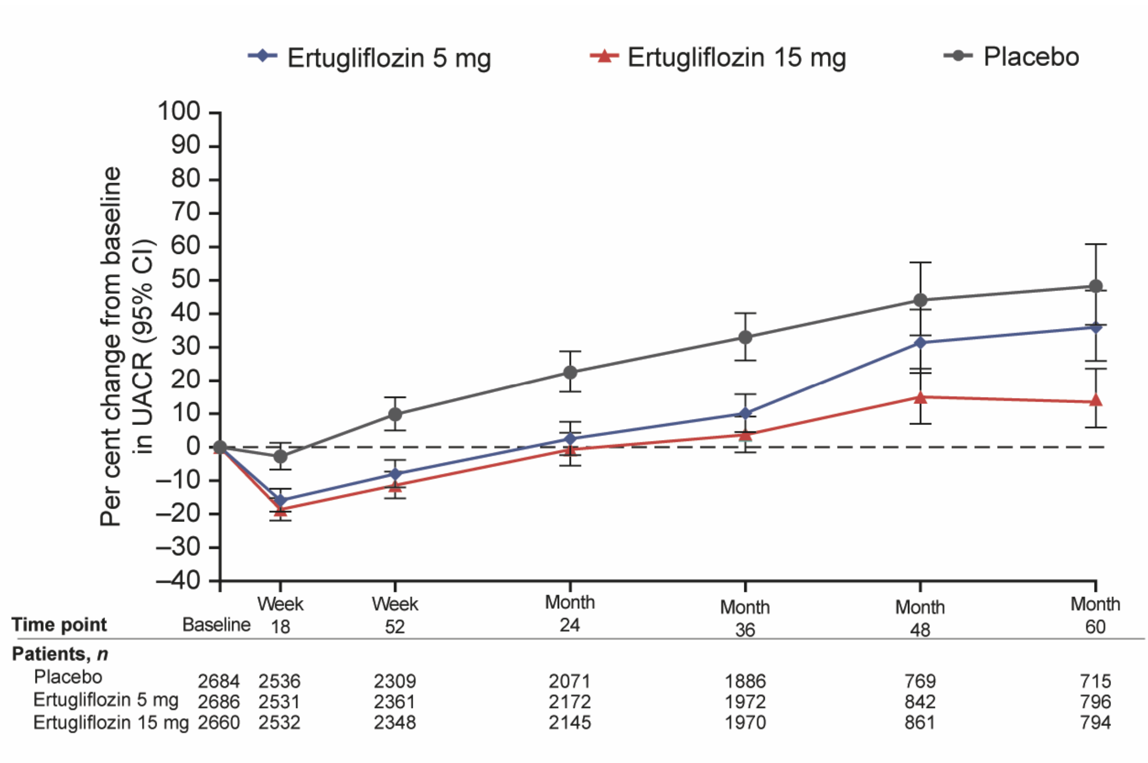

The number for treatment groups at each time point was the number of participants on study medication or who discontinued study medication ≤2 days before the sample was collected. Differences in per cent change from baseline (95% CI) relative to placebo at Week 18 were −13.5 (−18.3, −8.4) and −16.3 (−20.9, −11.4) in the ertugliflozin 5 mg and ertugliflozin 15 mg groups, respectively; at Week 52 these were −16.2 (−21.3, −10.8) and −19.3 (−24.2, −14.2) in the ertugliflozin 5 mg and ertugliflozin 15 mg groups, respectively; at Month 24 these were −16.4 (−21.9, −10.5) and −19.0 (−24.3, −13.2) in the ertugliflozin 5 mg and ertugliflozin 15 mg groups, respectively; at Month 36 these were −17.2 (−23.0, −11.0) and −22.1 (−27.5, −16.2) in the ertugliflozin 5 mg and ertugliflozin 15 mg groups, respectively; at Month 48 these were −9.3 (−18.2, 0.5) and −20.2 (−28.0, −11.6) in the ertugliflozin 5 mg and ertugliflozin 15 mg groups, respectively; at Month 60 these were −8.3 (−17.9, 2.5) and −23.5 (−31.5, −14.5) in the ertugliflozin 5 mg and ertugliflozin 15 mg groups, respectively.

FAS, full analysis set; UACR, urinary albumin-to-creatinine ratio.

Fig. 5 Per cent change from baseline in UACR in participants assigned to the KDIGO CKD low-risk category (**a**), moderate-risk category (**b**) or high-/very high-risk category (**c**) (all FAS)

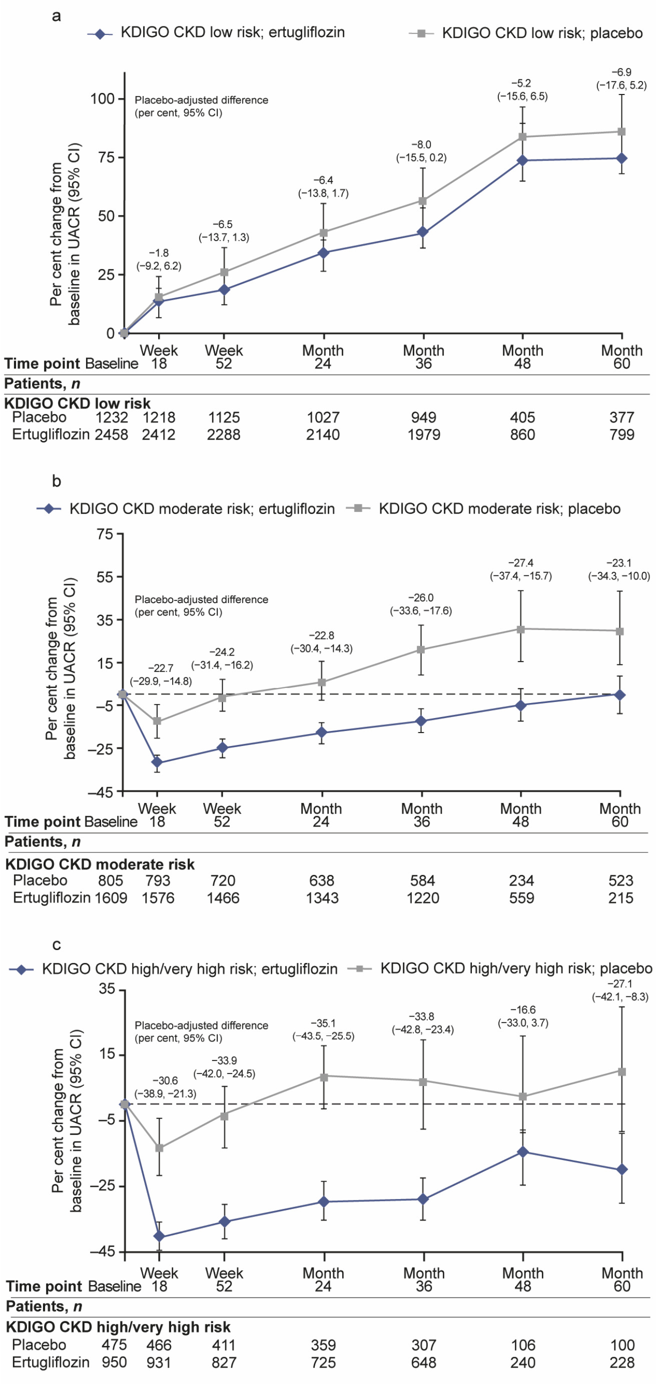

The number for treatment groups at each time point was the number of participants on study medication or who discontinued study medication ≤2 days before the sample was collected. FAS, full analysis set; KDIGO CKD, Kidney Disease Improving Global Outcomes chronic kidney disease; UACR, urinary albumin-to-creatinine ratio.

Fig. 6 Per cent change from baseline in UACR in participants with CKD stage 1 at baseline (**a**), CKD stage 2 at baseline (**b**) or CKD stage 3 at baseline (**c**) (all FAS)

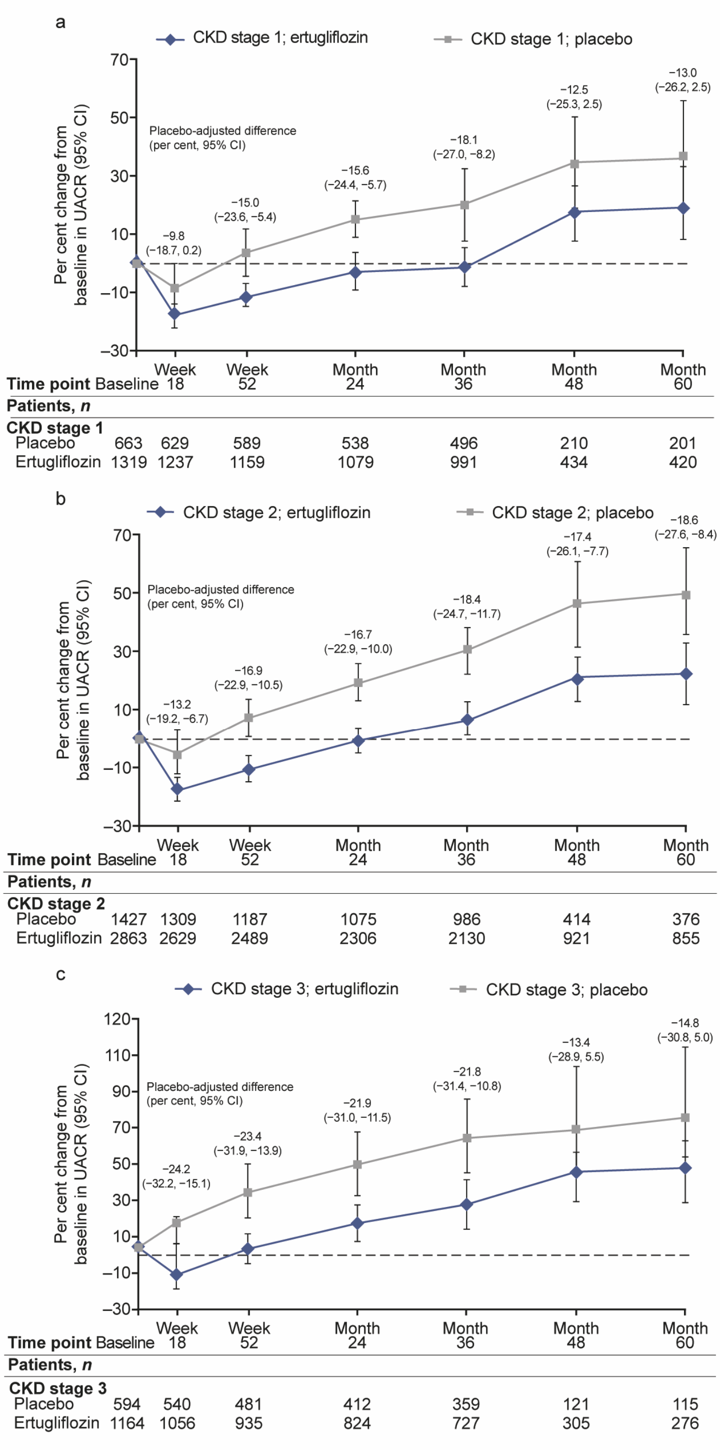

The number for treatment groups at each time point was the number of participants on study medication or who discontinued study medication ≤2 days before the sample was collected. FAS, full analysis set; KDIGO CKD, Kidney Disease Improving Global Outcomes chronic kidney disease; UACR, urinary albumin-to-creatinine ratio.

Fig. 7 Mean eGFR over time in the overall population using the MDRD equation (**a**), by ertugliflozin dose using the CKD-EPI equation (**b**) or by ertugliflozin dose using the MDRD equation (**c**) (all FAS)

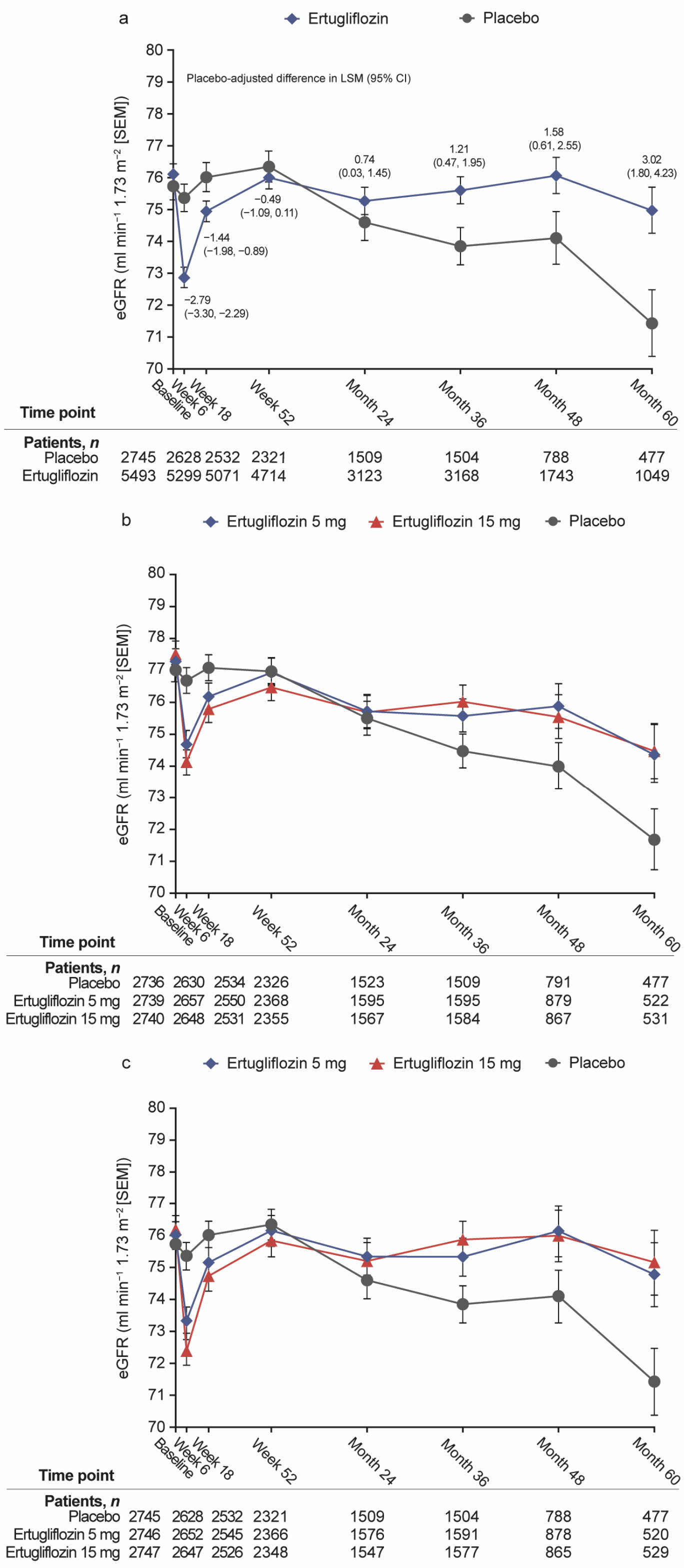

The number for treatment groups at each time point was the number of participants on study medication or who discontinued study medication ≤2 days before the sample was collected. Panel b: Differences in least squares mean (ml min^{-1} $[1.73\ m]^{-2}$ [95% CI]) relative to placebo at Week 6 were −2.16 (−2.64, −1.68) and −2.87 (−3.35, −2.39) in the ertugliflozin 5 mg and 15 mg groups, respectively; at Week 18 these were −1.16 (−1.70, −0.63) and −1.76 (−2.30, −1.23) in the ertugliflozin 5 mg and 15 mg groups, respectively; at Week 52 these were −0.01 (−0.61, 0.59) and −0.77 (−1.37, −0.17) in the ertugliflozin 5 mg and 15 mg groups, respectively; at Month 24 these were 0.66 (−0.05, 1.36) and −0.03 (−0.74, 0.68) in the ertugliflozin 5 mg and 15 mg groups, respectively; at Month 36 these were 1.03 (0.27, 1.78) and 0.94 (0.18, 1.70) in the ertugliflozin 5 mg and 15 mg groups, respectively; at Month 48 these were 1.76 (0.79, 2.72) and 1.41 (0.43, 2.38) in the ertugliflozin 5 mg and 15 mg groups, respectively; at Month 60 these were 2.56 (1.35, 3.77) and 2.54 (1.33, 3.75) in the ertugliflozin 5 mg and 15 mg groups, respectively. Panel c: Differences in least squares mean (ml min^{-1} $[1.73\ m]^{-2}$ [95% CI]) relative to placebo at Week 6 were −2.29 (−2.88, −1.70) and −3.30 (−3.89, −2.71) in the ertugliflozin 5 mg and 15 mg groups, respectively; at Week 18 these were −1.13 (−1.76, −0.50) and −1.74 (−2.38, −1.11) in the ertugliflozin 5 mg and 15 mg groups, respectively; at Week 52 these were −0.16 (−0.85, 0.54) and −0.83 (−1.53, −0.14) in the ertugliflozin 5 mg and 15 mg groups, respectively; at Month 24 these were 1.17 (0.36, 1.99) and 0.31 (−0.51, 1.13) in the ertugliflozin 5 mg and 15 mg groups, respectively; at Month 36 these were 1.24 (0.38, 2.09) and 1.18 (0.33, 2.04) in the ertugliflozin 5 mg and 15 mg groups, respectively; at Month 48 these were 1.70 (0.60, 2.79) and 1.49 (0.39, 2.59) in the ertugliflozin 5 mg and 15 mg groups, respectively; at Month 60 these were 3.01 (1.63, 4.39) and 3.05 (1.67, 4.43) in the ertugliflozin 5 mg and 15 mg groups, respectively. CKD, chronic kidney disease; CKD-EPI, Chronic Kidney Disease Epidemiology Collaboration; FAS, full analysis set; MDRD, Modification of Diet in Renal Disease; UACR, urinary albumin-to-creatinine ratio.

Fig. 8 Mean eGFR over time in participants assigned to the KDIGO CKD low-risk category (**a**), moderate-risk category (**b**) or high-/very high-risk category (**c**) (all FAS)

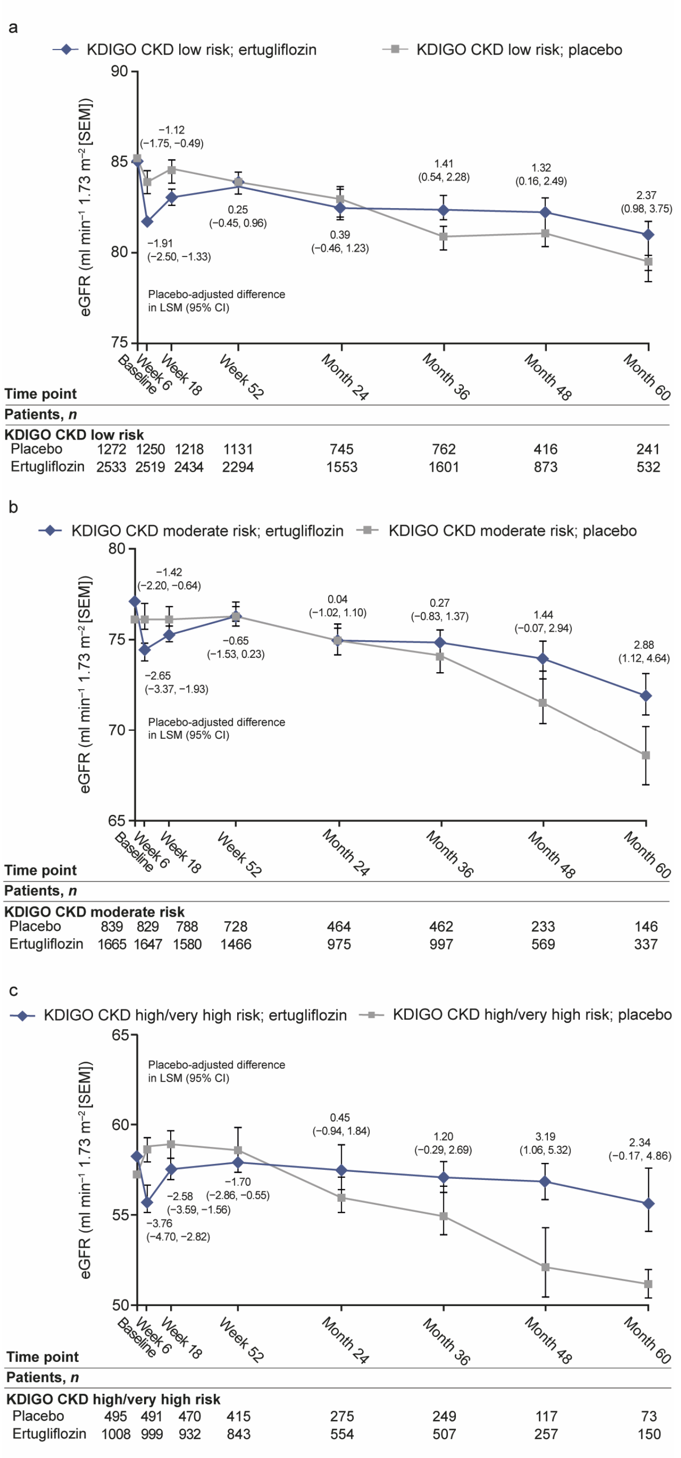

eGFR calculated using the CKD-EPI equation. The number for treatment groups at each time point was the number of participants on study medication or who discontinued study medication ≤2 days before the sample was collected. CKD-EPI, Chronic Kidney Disease Epidemiology Collaboration; FAS, full analysis set; KDIGO CKD,

Kidney Disease: Improving Global Outcomes chronic kidney disease; LSM, least squares mean; UACR, urinary albumin-to-creatinine ratio.

Fig. 9 Mean eGFR over time in participants with CKD stage 1 at baseline (**a**), CKD stage 2 at baseline (**b**) or CKD stage 3 at baseline (**c**) (all FAS)

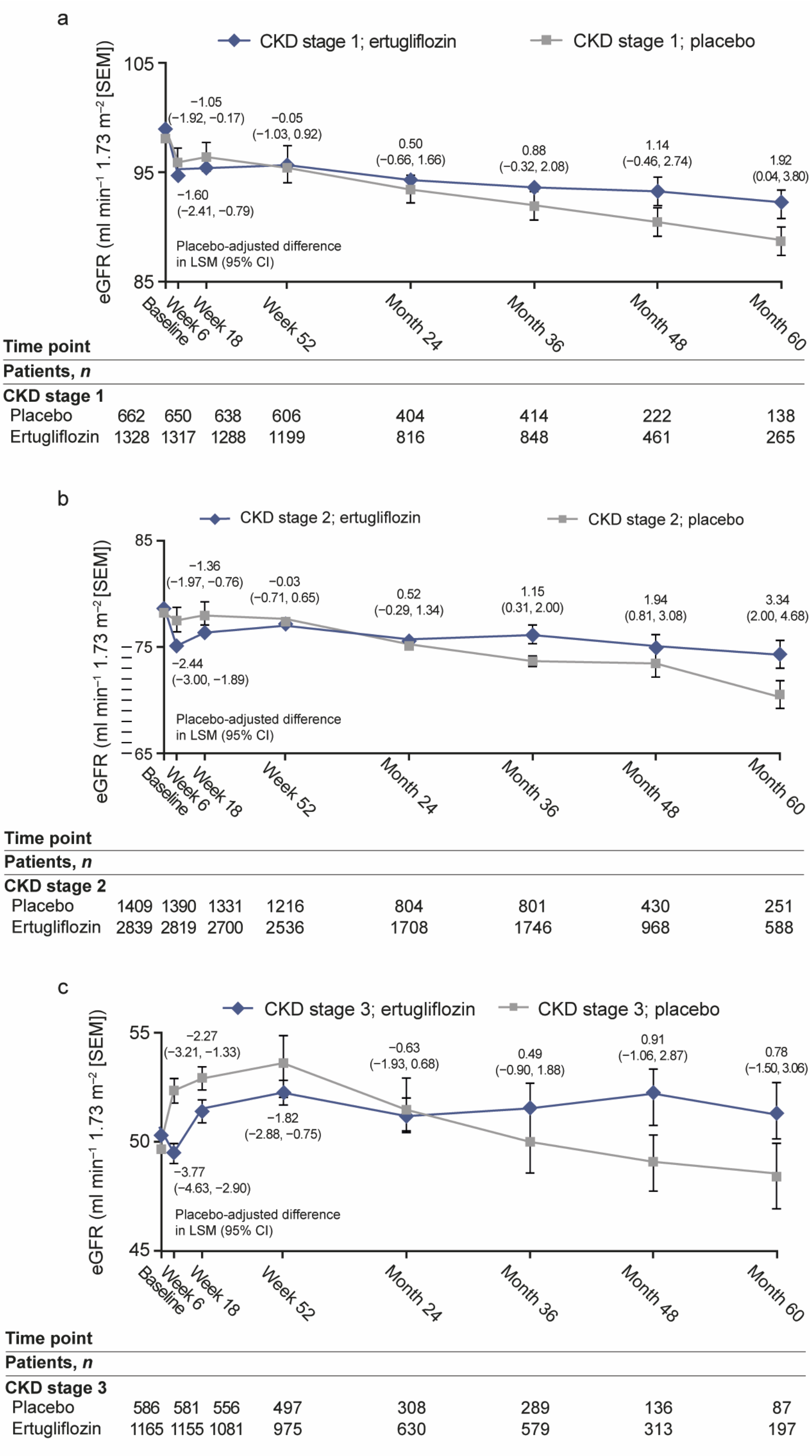

eGFR calculated using the CKD-EPI equation. The number for treatment groups at each time point was the number of participants on study medication or who discontinued study medication ≤2 days before the sample was

collected. CKD-EPI, Chronic Kidney Disease Epidemiology Collaboration; FAS, full analysis set; LSM, least squares mean; UACR, urinary albumin-to-creatinine ratio.
